# Current Advances on Nanomaterials Interfering with Lactate Metabolism for Tumor Therapy

**DOI:** 10.1002/advs.202305662

**Published:** 2023-11-08

**Authors:** Qian Cheng, Xiao‐Lei Shi, Qi‐Lin Li, Lin Wang, Zheng Wang

**Affiliations:** ^1^ Department of Clinical Laboratory Union Hospital Tongji Medical College Huazhong University of Science and Technology Wuhan 430022 China; ^2^ Research Center for Tissue Engineering and Regenerative Medicine Union Hospital Huazhong University of Science and Technology Wuhan 430022 China; ^3^ Hubei Key Laboratory of Regenerative Medicine and Multi‐disciplinary Translational Research Wuhan 430022 China; ^4^ Department of Gastrointestinal Surgery Union Hospital Tongji Medical College Huazhong University of Science and Technology Wuhan 430022 China

**Keywords:** glycolysis, lactate metabolism, nanoparticles, tumor therapy

## Abstract

Increasing numbers of studies have shown that tumor cells prefer fermentative glycolysis over oxidative phosphorylation to provide a vast amount of energy for fast proliferation even under oxygen‐sufficient conditions. This metabolic alteration not only favors tumor cell progression and metastasis but also increases lactate accumulation in solid tumors. In addition to serving as a byproduct of glycolytic tumor cells, lactate also plays a central role in the construction of acidic and immunosuppressive tumor microenvironment, resulting in therapeutic tolerance. Recently, targeted drug delivery and inherent therapeutic properties of nanomaterials have attracted great attention, and research on modulating lactate metabolism based on nanomaterials to enhance antitumor therapy has exploded. In this review, the advanced tumor therapy strategies based on nanomaterials that interfere with lactate metabolism are discussed, including inhibiting lactate anabolism, promoting lactate catabolism, and disrupting the “lactate shuttle”. Furthermore, recent advances in combining lactate metabolism modulation with other therapies, including chemotherapy, immunotherapy, photothermal therapy, and reactive oxygen species‐related therapies, etc., which have achieved cooperatively enhanced therapeutic outcomes, are summarized. Finally, foreseeable challenges and prospective developments are also reviewed for the future development of this field.

## Graphical abstract

Lactate has long been overlooked in tumors, confined to the roles of a glycolysis waste product and a biomarker of malignancy. With in‐depth research, lactate has recently been recognized as an important factor in tumor progression, metastasis, and therapeutic resistance. This review comprehensively summarizes the recent progress in nanomaterial‐mediated lactate metabolism modulation for oncotherapy.

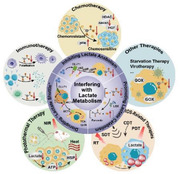

## 1 Introduction

Tumor, as one of the deadliest diseases in the world, seriously threatens human health and social development. High morbidity and mortality of tumors have driven a large number of researchers to find more effective approaches to fight against tumors. Up to now, diverse strategies have been developed to treat tumors, including surgery, chemotherapy, radiotherapy (RT), immunotherapy, gene therapy, phototherapy, and so on.^[^ ^1^ ^]^ Despite encouraging advancements, the adaptive evolution of tumor cells and the formation of protumor microenvironment greatly limit the therapeutic effect.^[^ ^2^ ^]^ That is, tumor plasticity is a winning strategy for tumor cells, allowing them to evolve stepwise to acquire adaptive phenotypes and survive under stressful conditions (e.g., chemotherapeutics drug treatment). In the process of evolution, tumor cells can alter and maintain their survival and development microenvironments through autocrine and paracrine, thus forming a protumor microenvironment to assist tumor growth and development.^[^ ^3^ ^]^ Therefore, it is of great significance to combat the adaptive phenotype of tumors and reverse the protumor microenvironment to improve the tumor therapeutic efficiency.

Lactate accumulation is an important hallmark of the tumor microenvironment (TME), which is a result of tumor metabolic adaptive evolution.^[^ ^4^ ^]^ To survive in the hypoxic and nutrient‐deficient TME and meet the energy demands of their uncontrolled growth, tumor cells shift glucose metabolism from oxidative phosphorylation (OXPHOS) to glycolytic pathway, which is known as the “Warburg effect”.^[^ ^5^ ^]^ According to the Warburg effect, tumor cells prefer to obtain energy through anaerobic glycolysis even in the presence of sufficient oxygen. This metabolic shift results in the accumulation of lactate in TME.^[^ ^5^ ^]^ Lactate in tumors has long been overlooked, confined to the role of a metabolic byproduct derived from glycolysis. With the deepening of tumor research, scientists have come to realize that lactate plays a central role in tumor progression, metastasis, and therapy resistance.^[^ ^6^ ^]^

The presence of both aerobic and hypoxic regions in solid tumors results in significant metabolic heterogeneity among different tumor cell populations.^[^ ^7^ ^]^ Typically, tumor cells in the hypoxic region consume glucose through anaerobic glycolysis and release lactate, which is then used by tumor cells in the adjacent aerobic tumor region as tricarboxylic acid (TCA) cycle intermediates. Tumor cells with different metabolic patterns cooperate with each other via the “lactate shuttle” to achieve metabolic symbiosis.^[^ ^8^ ^]^ This metabolic coupling established by lactate endows tumor cells with a metabolic competitive advantage in the nutrient‐deficient TME, maintaining rapid tumor growth.^[^ ^9^ ^]^ On the other hand, lactate involves in the formation of the protumor microenvironment, which affects the behaviors and functions of various cells in tumor tissues. High‐lactate microenvironments can enhance tumor cell motility and migration ability, as well as activate epithelial‐to‐mesenchymal transition, promoting tumor metastasis. The acidic TME caused by lactate accumulation can impair tumoricidal immune cell function and increase protumoral immunosuppressive cell infiltration, promoting tumor cell immune escape and tumor growth.^[^ ^10^ ^]^ Besides, the accumulation of lactate in TME is also closely associated with tumor tolerance.^[^ ^2^ ^]^ Thus, modulating tumor lactate metabolism is conducive to overcoming tumor adaptive evolution and resetting the protumor microenvironment, thereby improving the antitumor effect.

**Figure 1:**
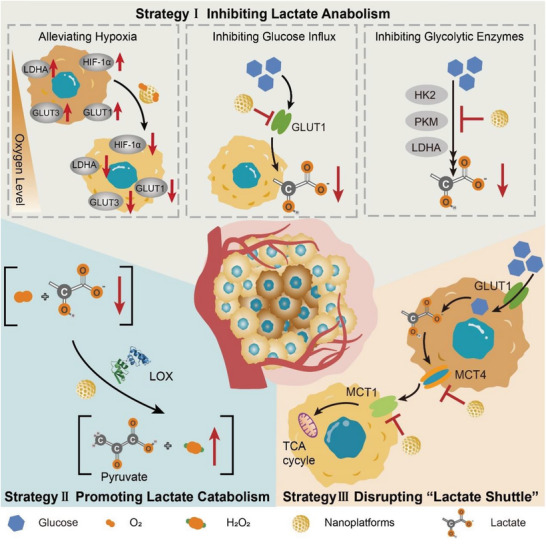
Schematic illustration of tumor treatment strategies based on nanomaterials interfering with lactate metabolism, including inhibiting lactate anabolism, promoting lactate catabolism, and disrupting “lactate shuttle”.

Nanomaterials hold great promise in tumor therapy due to their unique performance, including size advantages, optical properties, and catalytic activity.^[^ ^1a,b,d,e^ ^]^ Nanomedicines have an advantage over traditional pharmaceuticals in tumor treatment because they can prioritize drug delivery to the tumor sites, simultaneously provide multiple therapeutic agents for combination therapy, prolong drug‐circulation time, and manage drug‐release kinetics.^[^ ^11^ ^]^ Therefore, the past few decades have witnessed rapid progress in the synthesis of nanoparticles and the development of “smart” nanomaterials for tumor treatment.^[^ ^12^ ^]^ Recently, with the in‐depth study on tumor lactate metabolism, accumulating data have indicated that nanoparticles have great potential in targeted interference with tumor lactate metabolism. Notably, various nano antitumor therapies targeting lactate metabolism have initially achieved promising results, opening up a new path for tumor treatment. In this review, we systematically summarize the latest progress in nanotechnology for enhancing tumor therapy via interfering with tumor lactate metabolism. According to different processes of lactate metabolism regulated by various nanosystems, we classified these strategies as inhibiting lactate anabolism, promoting lactate catabolism, and disrupting the “lactate shuttle” (**Figure** **1**). Further, we discussed some typical examples of combining lactate metabolism modulation with other therapies (e.g., chemotherapy, immunotherapy, photothermal therapy (PTT), and reactive oxygen species (ROS)‐related therapies) to improve antitumor efficacy. Through a detailed summary and discussion of these strategies, we attempt to bring a comprehensive understanding of this field and provide some valuable guidance for designing nanosystems targeting lactate metabolism for tumor therapy.

## 2 Strategies of Nanomaterial‐Mediated Lactate Metabolism Modulation

### 2.1 Inhibiting Lactate Anabolism

**Figure 2:**
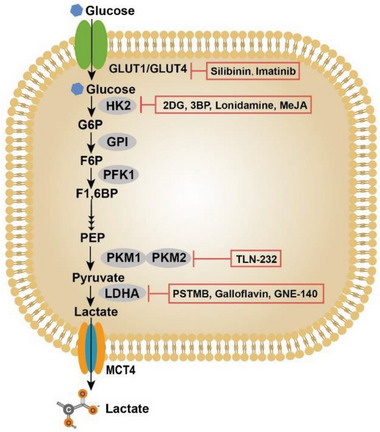
Lactate anabolism of tumor cells. Tumor cells are programmed to rely on aerobic glycolysis to support their proliferation. The abnormal metabolism of tumor cells causes a large accumulation of lactate in TME. Glucose transporters (i.e., GLUT1 and GLUT4) are responsible for the influx of glucose, which is then catalyzed by a series of glycolysis‐related enzymes to produce lactate. Following that, lactate is discharged into the extracellular environment through MCT4 on the cell membrane, leading to the increasement of lactate levels in TME. Enzymes that catalyze the metabolic reactions and corresponding inhibitors are shown in ovals and rectangles, respectively. GLUT1/GLUT4, glucose transporter 1/4; G6P, glucose‐6‐phosphate; F6P, fructose‐6‐phosphate; F1,6BP, fructose‐1,6‐bisphosphate; PEP, phosphoenolpyruvate; HK2, hexokinase 2; PFK1, phosphofructokinase 1; PKM1, pyruvate kinase 1; PKM2, pyruvate kinase 2; LDHA, lactate dehydrogenase A; MCT4, monocarboxylate transporter 4.

Inhibition of lactate anabolism can reduce lactate accumulation in TME at the source, damage tumor adaptability endowed by the metabolic switch, and thus inhibit the survival and development of tumors under severe conditions. Oncogenic lesions in tumors drive the metabolism switch into aerobic glycolysis to produce lactate by inducing the high expression and activation of glycolysis‐related enzymes. The hyperactive aerobic glycolysis in malignant tumor cells leads to “glucose addiction”.^[^ ^13^ ^]^ Therefore, tumor cells overexpress glucose transporters (GLUTs) to promote transmembrane transport of glucose for maintaining rapid proliferation.^[^ ^14^ ^]^ After being transported into tumor cells, glucose is converted to lactate under the catalytic action of a series of enzymes, including hexokinase 2 (HK2), glucose‐6‐phosphate isomerase, phosphofructokinase 1 (PFK1), pyruvate kinase isozyme M1 (PKM1), PKM2, and lactate dehydrogenase A (LDHA), etc. (**Figure** **2**). Thus, a direct blockage on the glycolysis processes of tumor cells by inhibiting glucose transport and deactivating glycolysis‐related enzymes can significantly reduce the lactate levels in TME (**Table** **1**).^[^ ^15^ ^]^ In addition, it is worth noting that some studies have shown that alleviating hypoxic TME can reverse tumor glucose metabolism patterns due to the metabolism plasticity of tumor cells, ultimately reducing the glycolysis level and lactate anabolism.^[^ ^16^ ^]^

**Table 1:** Summary of nanomaterial‐mediated lactate anabolism inhibition.

Strategies	Key nanomaterials	Targets	Functional agents	Refs.
Glucose influx inhibition	Polymer nanocarriers	GLUT1	Phloretin, DC, BAY‐876	[19, 25]
	Liposomal nanocarriers	GLUT1	Genistein	[19b]
	GNRs nanocarriers	GLUT1	DC	[26]
	Organosilica‐micelles	GLUT1	Genistein	[27]
	Sugar‐originated nanoparticles	GLUT1	sCND	[32]
	UCNP@MnO_3‐_ *_X_*	GLUT1	ROS	[31]
Hypoxia alleviation	Polymer nanocarriers	HIF‐α, GLUT1, HK2, PKM2, LDHA	Gefitinib and YAP‐siRNA, miR‐519c	[20, 198]
	Fluorocarbon‐based nanocarriers	HIF‐α, GLUT1, HK2	O_2_	[41, 42]
	Metal and metal oxide/complex nanozymes	HIF‐α, GLUT1, LDHA	O_2_	[20, 47, 77]
	MnO_2_	HIF‐α, GLUT1	O_2_	[48, 50]
	Thylakoid‐based nanoparticles	HIF‐α, GLUT	O_2_	[52]
Glycolytic enzymes inhibition	Polymer nanocarriers	HK2, PKM2, PDK1, LDHA, PDK1, GADPH, PFKFB4,^a)^ PDK2	LND, siPKM2, DCA, ZM1068‐NB, boric acid, siPFKFB4, siLDHA, shHK2, shPDK2, JX06, 2‐DG	[15, 52, 66, 72, 77, 79, 80, 90, 199]
	Liposomal nanocarriers	HK2, PKM2, LDHA, PFKFB3^b)^	2‐DG, shikonin, shLDHA, siLDHA, PFK15	[15, 59, 77, 78, 161, 174]
	nMOFs	PKM2, PFKFB3, HK2, LDHA	siPKM2, PFK15, 2‐DG, GSK2837808A (GSK)	[15, 73, 77, 165]
	MSN	HK2, PFK1, PKM2	EDTA	[21, 86]
	Lactoferrin‐based nanocarriers	PKM2	Shikonin	[66, 70]
	Au nanoparticles	HK2	3‐BP, siHK2	[57, 59]
	MnO_2_	HK2	2‐DG	[59d]
	CCN nanozyme	PKM2	CCN	[74]
	Cell microparticles	HK2	3‐BP	[59a]
	Selenium nanoparticles	HK2, PK	SeNPs	[88]
	Self‐assembled nanodrugs	HK2, PKM2, LDHA, LDHB	Adjudin, glycopeptide, DCA, α‐lactalbumin‐oleic acid	[21, 77, 200]
	Zinc‐enriched Prussian blue	LDHA, HK2	Zn^2+^, LND	[30]
	Supramolecular nanoplatforms	HK2, c‐Myc, LDHA	3‐BP, JQ1, LND	[63, 77, 201]

^a)^ 6‐Phosphofructo‐2‐kinase/fructose‐2,6‐bisphosphatase 4

^b)^ 6‐Phosphofructo‐2‐kinase/fructose‐2,6‐bisphosphatase 3.

Currently, some drugs targeting GLUTs and glycolytic enzymes have been extensively studied, such as GLUT1 inhibitors (silibinin and imatinib), HK2 inhibitors (2‐deoxy‐d‐glucose (2‐DG), 3‐bromopyruvate (3‐BP), and lonidamine (LND)), LDHA inhibitors (PSTMB, GNE‐140), etc.^[^ ^17^ ^]^ (**Table** **2**). However, the clinical translation of these agents is greatly challenged by their considerable systemic toxicity and undesirable therapeutic efficiency. The inherent physicochemical properties of nanoparticles, such as tumor targeting, stability in blood circulation, regulation ability to enzyme activity, and controllable oxygen production ability, make them suitable for the targeted inhibition of tumor cell glycolysis.^[^ ^18^ ^]^ Given this, researchers have developed a series of nanoplatforms to inhibit lactate production by tumor cells through different intervention strategies, including inhibiting glucose transport,^[^ ^19^ ^]^ alleviating tumor hypoxia,^[^ ^20^ ^]^ and reducing glycolysis‐related enzyme activities.^[^ ^15^, ^21^ ^]^ These nanosystems have been shown to interfere with tumor cell energy metabolism and reverse the protumor microenvironment to effectively restrain tumor growth.

**Table 2:** Representative preclinical and clinical studies for lactate‐related strategies.

Strategy^a)^	Drugs	Target	Identifier^b)^	Research phase	Tumor types	Refs.
Lactate anabolism inhibition	WZB117, fasentin, RNAi, glutor, silybin, BAY‐876, phloretin	GLUT1	–	Preclinical	–	[24, 25, 202]
	Silibinin	GLUT1	NCT05689619, NCT00487721	I/II	NSCLC, breast cancer	–
	Genistein	GLUT1	NCT01126879, NCT00244933, NCT01985763, etc.	I/II	Prostate cancer, breast cancer, colorectal cancer, etc.	–
	Apigenin	GLUT1	NCT00609310	II	Colorectal cancer	–
	Ritonavir	GLUT1/4	NCT01009437, NCT05679388, NCT05242926	I	Breast cancer, prostate cancer, solid tumor of adult	–
	3‐BP, lonidamine, methyl jasmonate (MeJA), RNAi	HK2	–	Preclinical	–	[203]
	Benserazide, alkannin, DASA‐58, oleanolic acid (OA), dimethylaminomicheliolide, resveratrol	PKM2	–	Preclinical	–	[64, 67, 204]
	GNE‐140, FK866, galloflavin, AT101, FX‐11, *N‐*hydroxy‐2‐carboxysubstituted indoles, PSTMB	LDH	–	Preclinical	–	[205]
	3PO, PFK15	PFK	–	Preclinical	–	[120, 161]
	2‐DG	HK2	NCT00096707, NCT00633087	I/II	Solid malignancy, hormone refractory prostate cancer	–
	Shikonin	PKM2	NCT01968928	Observational	Urothelial carcinoma	–
	TLN‐232	PKM2	NCT00735332	II	Melanoma	–
	AT‐101	LDH	NCT00390403, NCT00286780, NCT00397293, etc.	I/II	Central nervous system tumors, chronic lymphocytic leukemia, SCLC, etc.	–
	DCA	PDK1	NCT01111097	I	Brain tumors	–
“Lactate shuttle” inhibition	α‐CHC, RNAi, BAY‐8002, AR‐C155858, AR‐C117977	MCT1	–	Preclinical	–	[143, 205, 206]
	AZD3965	MCT1	NCT01791595	I	Advanced cancer	–
	Diclofenac	MCT1/4	NCT01935531	IV	Basal cell carcinoma	–
	AZ93, lonidamine	MCT4	–	Preclinical	–	[21, 207]
	Fluvastatin	MCT4	NCT02115074	I	Optico‐chiasmatic gliomas	–

^a)^ Strategies of lactate catabolism are currently in the laboratory research stage, and LOX is the main therapeutic agent

^b)^ Clinical data come from ClinicalTrials. gov.

#### 2.1.1 Inhibiting Glucose Transport

GLUTs are a group of membrane‐associated carrier proteins responsible for transporting hydrophilic glucose into cells through hydrophobic membranes. Among them, GLUT1 is the most widely distributed transporter in tissues serving as the primary carrier for glucose transport.^[^ ^22^ ^]^ Numerous studies have shown that GLUT1 is upregulated in various tumors, including renal cancer, lung cancer, and colorectal cancer, ensuring that glucose is efficiently transported into tumor cells to meet the surging nutritional demand.^[^ ^23^ ^]^ Given the critical role of GLUT1 in tumor development, inhibition of GLUT1 holds a promising prospect in tumor therapy.

**Figure 3:**
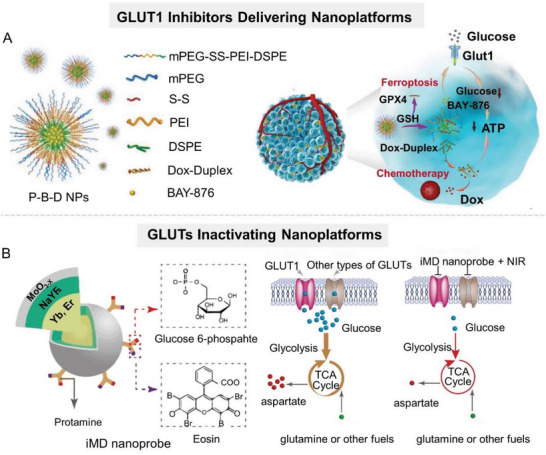
Nanomaterial‐mediated lactate anabolism inhibition by blocking glucose transport. A) Schematic illustration of P‐B‐D NPs, which are fabricated by coloading GLUT1 inhibitor (BAY‐876) and DOX prodrug (DOX‐Duplex) into a GSH‐responsive self‐assembled polymer nanoparticle. Reproduced with permission.^[^ ^25^ ^]^ Copyright 2021, Wiley‐VCH. B) Schematic diagram of iMD nanoparticle that inactivates multiple types of GLUTs on tumor cell membranes, which is composed of a core–shell UCNP with a thin layer of MnO_3_‐*_X_*, protamine, photosensitizer eosin, and glucose phosphate. Reproduced with permission.^[^ ^31^ ^]^ Copyright 2021, American Chemical Society.

To date, many small‐molecule GLUT1 inhibitors have been developed, such as WZB117, BAY‐876, silibinin, etc.^[^ ^24^ ^]^ To improve the therapeutic efficacy and reduce side effects, researchers have developed a variety of nanocarriers, including polymer nanocarriers,^[^ ^19^, ^25^ ^]^ liposomes,^[^ ^19^ ^]^ gold nanorods (GNRs),^[^ ^26^ ^]^ and organosilica‐micelles,^[^ ^27^ ^]^ for the targeted delivery of GLUT1 inhibitors to tumor sites. GLUT1 inhibitors are loaded into these nanocarriers through different mechanisms, including hydrophobic interactions, electronic interactions, π–π interactions, etc. These nanocarriers have been shown to improve the efficacy of GLUT1 inhibitors to some extent. However, the metabolic plasticity of tumor cells still leads to the limited efficacy of GLUT1 inhibitors.^[^ ^28^ ^]^ It is well known that one advantage of nanotherapeutics is their ability to integrate multiple therapeutic components into a single platform.^[^ ^29^ ^]^ Therefore, researchers often load GLUT1 inhibitors on nanocarriers along with other therapeutic drugs to improve the overall therapeutic effect. For example, Jiang et al. coloaded GLUT1 inhibitor (BAY‐876) and doxorubicin prodrug (DOX‐Duplex) into a glutathione (GSH)‐responsive self‐assembled polymer nanocarrier (denoted as P‐B‐D) to achieve controlled release of different functional agents in tumor site (**Figure** **3**A).^[^ ^25^ ^]^ After enrichment to the tumor site via the enhanced permeability and retention (EPR) effect, the nanosystem disintegrated and released BAY‐867 and DOX‐Duplex attributed to the breakage of disulfide bonds in response to the overexpressed GSH in TME. Both in vitro and in vivo experimental data demonstrated that this dual‐drug delivery nanosystem significantly inhibited the energy metabolism of tumor cells, and exhibited a stronger tumor suppressive effect than the nanosystem with BAY‐867 or Dox‐Duplex alone. While multidrug delivery nanosystems can improve antitumor efficacy, they are also more complex and difficult to prepare. Typically, when multiple chemotherapeutic drugs are coloaded on one nanoplatform, differences in solubility, stability, lipophilicity, charge, and other properties of the different drugs must be taken into account. Compared with coloading multiple drugs, elaborately combining the inherent properties of nanoparticles to kill tumor cells would be more conducive to simplifying nanosystems to achieve efficient antitumor effects. Some nanoparticles, such as GNRs^[^ ^26^ ^]^ and Prussian blue,^[^ ^30^ ^]^ can not only serve as high‐performance drug carriers but also convert light energy into heat that can induce irreversible apoptosis of tumor cells. With this in mind, Zhang's group developed a hyaluronic acid (HA)‐modified GNRs nanosystem (GNR/HA‐DC) for targeted delivery of the GLUT1 inhibitor (diclofenac, DC) to tumor sites.^[^ ^26^ ^]^ GNR/HA‐DC could be preferentially internalized by tumor cells because of the tumor‐targeting ability of HA and further released DC triggered by high‐level hyaluronidase (HAase) in tumor cells. The experimental results revealed that GNR/HA‐DC downregulated GLUT1 expression to inhibit the glycolysis process and generated heat to damage tumor cells under near‐infrared light, demonstrating an excellent tumor inhibition effect in vitro and in vivo.

Given the growing awareness of the ability of nanoparticles to directly regulate cellular biological processes, some nanoparticles have been shown to directly inhibit glucose uptake by tumor cells apart from serving as GLUT inhibitor carriers. Glucose, as the substrate of GLUT1, possesses the advantages of easy chemical functionalization, good biocompatibility, and nontoxicity, which allow it to serve as a GLUT1‐targeting ligand. Over the years, the development of glucose‐functionalized structures for targeted delivery of antitumor drugs and imaging agents to tumor cells has been one of the research focuses. Some studies have employed glucose molecules to functionalize nanoparticles to specifically interfere with the function of GLUTs. For example, Duan et al. designed an integrated molecular deactivator (iMD) based on an upconversion nanoparticle (UCNP) capable of inactivating multiple types of GLUTs on tumor cell membranes when exposed to 980 nm illumination (Figure 3B).^[^ ^31^ ^]^ iMD was constructed by coating with a thin layer of molybdenum oxide (MnO_3‐_ *_X_*) on the surface of UCNP and further modified with protamine, photosensitizer eosin, and glucose phosphate. The glucose phosphate on iMD could specifically recognize diverse GLUT isoforms on tumor cell membranes. When exposed to 980 nm laser light, iMD produced singlet oxygen species (^1^O_2_) via eosin, inactivating GLUTs that interacted with iMD. Experimental data showed that iMD precisely inactivated GLUTs to inhibit tumor cell proliferation in vivo. In addition to glucose molecules, sugar‐derived nanoparticles have also been demonstrated to have similar recognition and binding abilities for GLUTs. For this, Wang et al. developed a sugar‐derived carbon nanodot (sCND) that competed with glucose to enter tumor cells via the same transporter (GLUTs), resulting in a reduced glucose influx.^[^ ^32^ ^]^ Meanwhile, the sCND cannot be phosphorylated for the subsequent glycolysis, thereby reducing the energy supply of tumor cells.

In addition, some studies have focused on the targeted delivery of glucose oxidase (GOX) to tumor sites via nanoparticles to specifically consume extracellular glucose for glycolytic metabolism reduction.^[^ ^33^ ^]^

#### 2.1.2 Relieving Hypoxia

Due to the insufficient blood supply and rapid proliferation of tumor cells, solid tumors often suffer from severe hypoxic, in which hypoxia‐inducible factor‐1 (HIF‐1) is one of the most important hypoxia‐responsive proteins and serves as an index to measure tumor hypoxia.^[^ ^34^ ^]^ HIF‐1, composed of an O_2_‐sensitive α‐subunit (HIF‐1α) and a constitutively expressed β‐subunit (HIF‐1β), has been widely reported to be overexpressed in many malignancies, including breast cancer, gastric cancer, and colorectal cancer. Under hypoxic conditions, HIF‐1α is stabilized in the cytoplasm and then translocated into nuclear, where it heterodimerizes with HIF‐1β to form HIF‐1 transcription factor complexes that activate the expression of genes encoding glucose transporters (GLUT1, GLUT3, etc.) and glycolytic enzymes (HK2, LDHA, etc.),^[^ ^34^, ^35^ ^]^ consequently accelerating the anaerobic glycolysis and lactate accumulation in TME.^[^ ^35^, ^36^ ^]^ Given the metabolic plasticity of tumor cells, alleviating hypoxia in TME is expected to reverse the glucose metabolic patterns, and ultimately reduce glycolysis level and lactate anabolism. To date, researchers have developed a variety of functional nanomaterials for alleviating hypoxia within solid tumors, including “oxygen‐carrying” nanomaterials and “oxygen‐generating” nanomaterials.

**Figure 4:**
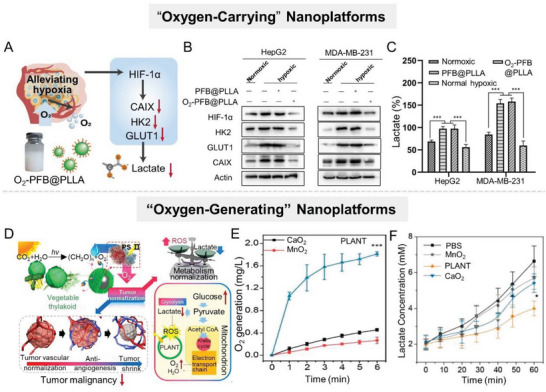
Alleviating hypoxia in TME to inhibit lactate anabolism. A) Scheme illustration of an “oxygen‐carrying” nanoplatform (PFB@PLLA) to downregulate glycolysis level and lactate secretion by alleviating hypoxia. B) Western blot analysis of HIF‐1α, HK2, GLUT1, and CAIX protein expressions. C) Lactate production of HepG2, MDA‐MB‐231 after different treatments. Reproduced with permission.^[^ ^42^ ^]^ Copyright 2019, Wiley‐VCH. D) Schematic design of the PLANT nanosystem fabricated by vegetable thylakoid and various nanoparticles. Under light irradiation, PLANT could produce O_2_ in situ to normalize TME for antiangiogenesis therapy and enhanced PDT. E) O_2_ generation capacity of different groups in real‐time. F) Lactate concentration of different groups in real‐time. Reproduced with permission.^[^ ^52^ ^]^ Copyright 2018, American Chemical Society.

“Oxygen‐carrying” nanomaterials are competent in delivering oxygen into tumors and releasing it in response to specific stimuli, while the “oxygen‐generating” nanomaterials can self‐decompose or catalyze other substrates to yield oxygen in specific conditions (e.g., low pH, hydrogen peroxide (H_2_O_2_), etc.), both of which facilitate the oxygen supply in tumors. These two types of nanomaterials have attracted considerable attention in terms of improving antitumor efficacy.^[^ ^37^ ^]^ “Oxygen‐carrying” nanomaterials should possess a high oxygen capacity as well as stably confine oxygen before reaching tumor sites. At present, the developed “oxygen‐carrying” nanomaterials include hemoglobin,^[^ ^38^ ^]^ fluorocarbon,^[^ ^39^ ^]^ metal–organic frameworks (MOFs),^[^ ^40^ ^]^ etc. It has been reported that “oxygen‐carrying” nanomaterials can downregulate the expression of GLUT1 by alleviating tumor hypoxia.^[^ ^41^ ^]^ For example, Chen et al. successfully constructed an “oxygen‐carrying” nanoplatform (PFB@PLLA) capable of releasing O_2_ in response to ultrasound stimulus by encapsulating methoxy poly(ethylene glycol)‐*b*‐poly(l‐lactide) (mPEG‐PLLA) shell onto liquid pentafluorobutane (PFB) core (**Figure** **4**A).^[^ ^42^ ^]^ Experiments demonstrated that PFB@PLLA released a large amount of oxygen at the tumor site with ultrasound stimulus, effectively downregulating hypoxia‐related proteins (HIF‐1 and carbonic anhydrase IX (CAIX)) and glycolysis‐related proteins (HK2 and GLUT1), and thereby reducing lactate production (Figure 4B,C).

“Oxygen‐generating” nanomaterials are a class of materials that can induce oxygen‐production reactions in the TME (with overexpressed H^+^ and H_2_O_2_), mainly including self‐decomposition oxygen‐production (CaO_2_,^[^ ^43^ ^]^ Au_2_O_3_,^[^ ^44^ ^]^ etc.) and catalytic oxygen‐production (MnO_2_,^[^ ^37^ ^]^ Prussian blue,^[^ ^37^, ^45^ ^]^ carbon nitride (C_3_N_4_),^[^ ^46^ ^]^ Fe_3_O_4_,^[^ ^37^ ^]^ noble metal,^[^ ^47^ ^]^ etc.). Due to their good biocompatibility and strong catalytic activity, MnO_2_ nanoparticles have recently become one of the most attractive materials for relieving tumor hypoxia.^[^ ^37^ ^]^ MnO_2_ nanoparticles can catalyze the decomposition of endogenous H_2_O_2_ to generate O_2_ in the acidic TME.^[^ ^48^ ^]^ However, it is worth noting that the O_2_ production capacity of MnO_2_ is severely limited by the insufficient H_2_O_2_ concentration in the TME, with endogenous concentrations ranging from 50 to 200 µM.^[^ ^49^ ^]^ To compensate for the insufficient substrate concentration, several strategies have been developed to elevate the H_2_O_2_ level in TME, thereby promoting the localized oxygen‐production reaction. Typically, Zhang's group fabricated a cascade catalytic nanoplatform (MG/HA) by assembling MnO_2_, GOX, and the tumor‐targeting polymer HA.^[^ ^50^ ^]^ GOX was capable of catalyzing the oxidation of glucose to produce H_2_O_2_ and gluconic acid, elevating the H_2_O_2_ concentration in the TME.^[^ ^51^ ^]^ Subsequently, MnO_2_ catalyzed the H_2_O_2_ decomposition under the acidic condition to generate abundant O_2_, thereby effectively relieving tumor hypoxia. Experiments revealed that this MG/HA not only depleted glucose but also downregulated GLUT1 expression, thereby inhibiting glucose metabolism for effective tumor suppression. In addition, inspired by the oxygenic photosynthetic of leaf cells, this group constructed a photoactivated oxygen‐producing nanosystem (PLANT) by coating thylakoid membranes on nanoparticles (Figure 4D).^[^ ^52^ ^]^ Compared with conventional oxygen‐producing materials, such as CaO_2_ and MnO_2_, the PLANT provided a higher oxygen generation efficiency under 660 nm laser irradiation (Figure 4E). Experimental results showed that the PLANT downregulated HIF‐1α expression and reduced the glucose consumption and lactate production rate (Figure 4F), thus reversing the metabolic patterns of tumor cells.

Although improving tumor oxygenation is expected to alter tumor metabolic patterns and reduce lactate anabolism to some extent, such indirect strategies often present limited regulatory effects on glucose metabolism. It should not be ignored that a large number of studies have demonstrated that alleviating hypoxia is conducive to overcoming the physiological barriers related to treatment tolerance in solid tumors and significantly enhances antitumor effects.^[^ ^53^ ^]^ This is mainly because the efficacy of many antitumor therapies, including photodynamic therapy (PDT),^[^ ^54^ ^]^ RT,^[^ ^55^ ^]^ and chemotherapy,^[^ ^56^ ^]^ depend largely on the oxygen level at the tumor site.

#### 2.1.3 Inhibiting Glycolysis‐Related Enzymes

The glycolysis pathway comprises a succession of enzymatic reactions involved with various glycolytic enzymes. Oncogene lesions shift the metabolic patterns of tumors to aerobic glycolysis with enhanced lactate generation through upregulation and activation of glycolytic enzymes. Thus, targeting these enzymes may elicit profound effects on tumor progression through the inhibition of glycolysis and lactate production (Figure 2).^[^ ^17^, ^57^ ^]^

**Figure 5:**
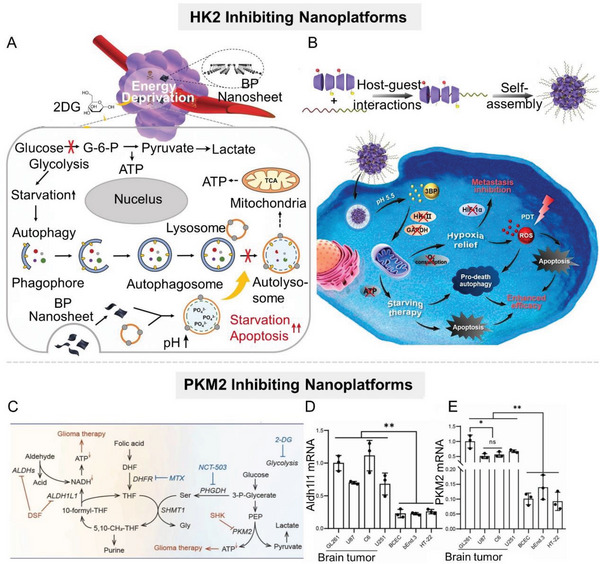
Nanomaterial‐mediated lactate anabolism inhibition by inhibiting glycolysis‐related enzymes HK2 and PKM2. A) Scheme of the augmenting tumor‐starvation strategy by combining autophagy inhibitors (BP nanosheet) and HK2 inhibitors (2‐DG). B) Scheme of the supramolecular nanoplatform (CD‐Ce6‐3BP) combining 3‐BP with Ce6 for downregulating HK2 and GAPDH, which can inhibit lactate anabolism, and induce tumor cell apoptosis. Reproduced with permission.^[^ ^63^ ^]^ Copyright 2020, American Chemical Society. C) Schematic diagram of the mechanism of the hybrid albumin/lactoferrin nanosystem (BSA/LF), which codelivers PKM2 inhibitor SHK and ALDH1L1 inhibitor DSF to achieve dual restraint of glioma energy metabolism. D,E) Detection of (D) ALDH1L1 and (E) PKM2 expressions in normal cell lines and glioma cell lines by qPCR (*n* = 3). Reproduced with permission.^[^ ^70^ ^]^ Copyright 2022, Wiley‐VCH.

The phosphorylation of glucose to glucose‐6‐phosphate (G‐6‐P) is the first rate‐limiting step in glycolysis, which is catalyzed by tissue‐specific isoenzyme HK2.^[^ ^17^ ^]^ Small‐molecule inhibitors such as 3‐BP and 2‐DG can block glycolysis progress at the initial stage by inhibiting the activity of HK2, resulting in tumor cell energy depletion and lactate production decrement, and ultimately inhibiting tumor growth.^[^ ^58^ ^]^ Some investigations employed nanocarriers to deliver small‐molecule inhibitors of HK2 to tumor sites for tumor treatment.^[^ ^59^ ^]^ However, the antitumor activity of glycolytic inhibitors may be greatly diminished by a cytoprotective mechanism called autophagy. To override energy metabolic deficiencies when glycolysis is inhibited, tumor cells boost the level of autophagy to “eat” and “digest” their cytoplasmic components, providing additional metabolic support for re‐establishing intracellular homeostasis, allowing tumor cells to survive in adverse conditions such as starvation.^[^ ^60^ ^]^ Given this, Shi's group developed a synergetic strategy by employing black phosphorus (BP) nanosheets to cut off the autophagic flux and compensatory energy supply of tumor cells, hence synergistically enhancing antiglycolytic agent‐induced starvation treatment (**Figure** **5**A).^[^ ^61^ ^]^ Briefly, the glycolysis pathway of tumor cells was blocked by 2‐DG, which acts as a glucose analog to be phosphorylated by HK2 to 2‐deoxy‐d‐glucose‐6‐phosphate (2‐DG‐6‐P) that cannot be further metabolized into lactate. Concurrently, the degradation of BP nanosheets produced a large amount of alkaline PO_4_^3−^, which depleted intracellular H^+^ and inhibited lysosomal functions, ultimately inhibiting the autophagy of tumor cells. This synergetic strategy showed remarkable antineoplastic effects both in vitro and in vivo. Notably, autophagy is a “double‐edged sword,” as the overactivation of autophagy would lead to autophagic cell death.^[^ ^62^ ^]^ For this, Deng et al. constructed a supramolecular nanoplatform (CD‐Ce6‐3BP) capable of converting the character of autophagy from prosurvival to prodeath via the combination of HK2 inhibitor (3‐BP) and photosensitizer chlorin e6 (Ce6) (Figure 5B).^[^ ^63^ ^]^ This supramolecular nanoplatform could efficiently improve the solubility of therapeutic agents, enhance the enrichment of agents at tumor sites, and enable the simultaneous delivery of different agents. When CD‐Ce6‐3BP nanosystem reached tumor sites, 3‐BP‐mediated starvation‐induced autophagy and Ce6‐mediated ROS‐induced autophagy were coupled to trigger excessive autophagy, boosting tumor cell apoptosis. Experimental results demonstrated that CD‐Ce6‐3BP downregulated the expression of HK2 and glyceraldehyde‐3‐phosphate dehydrogenase (GAPDH), inhibited lactate anabolism, and effectively induced tumor cell apoptosis.

PKM2 is a key rate‐limiting enzyme in the glycolysis process of tumor cells, which catalyzes the conversion of phosphoenolpyruvate (PEP) to pyruvate with the production of ATP. In recent years, PKM2 has been demonstrated to be overexpressed in a variety of cancers, including glioma, liver cancer, nonsmall cell lung cancer, melanoma, and cervical cancer,^[^ ^64^ ^]^ promoting tumor development and progression. PKM2 can then regulate tumor cell glucose metabolism via a feedback loop of c‐Myc, let‐7a, and hnRNPA1.^[^ ^65^ ^]^ Therefore, pharmacologically inhibiting PKM2 protein kinase activity by small‐molecule inhibitors (e.g., shikonin (SHK),^[^ ^66^ ^]^ benserazide,^[^ ^64^ ^]^ resveratrol,^[^ ^67^ ^]^ etc.) or silencing PKM2 gene by siRNA^[^ ^68^ ^]^ offer potential therapeutic strategies for certain tumors. However, the successful clinical translation of PKM2 inhibitors has not yet been achieved, mainly due to their systemic toxicity and the presence of alternative energy‐producing pathways in tumor cells. For example, NADH, a reduced form of nicotinamide‐adenine dinucleotide, can be transformed to ATP in tumor cells to support tumor progression as an alternative energy source, even when the glycolysis pathway is blocked.^[^ ^69^ ^]^ Zhao et al. demonstrated that 10‐formyl‐tetrahydrofolate‐NADH‐ATP metabolic enzyme ALDH1L1 and glycolytic enzyme PKM2 are overexpressed in glioma. Based upon this, the team designed a hybrid albumin/lactoferrin nanoparticle (BSA/LF NP) to codeliver PKM2 inhibitor SHK and ALDH1L1 inhibitor disulfiram (DSF) to achieve dual restraint of glioma energy metabolism (Figure 5C).^[^ ^70^ ^]^ Of note, the specific targeting of albumin and lactoferrin on glioma allowed the nanoparticle to efficiently penetrate the blood‐brain barrier and deliver therapeutic drugs to the tumor site. Experimental results showed that this nanoparticle inhibited glycolysis and the alternative energy‐producing pathway in tumor cells simultaneously, exhausting ATP to induce tumor cell apoptosis (Figure 5D,E). Although the research and development of siRNAs started later than conventional small‐molecule inhibitors, they exhibit more prominent advantages in therapeutic efficiency, safety, and long‐term efficacy, attracting the attention of many researchers. Therefore, targeting PKM2 genes with siRNA can largely compensate for the deficiency of small‐molecule inhibitors. However, unsatisfactory distribution, serious off‐target effects, and poor stability are three major “pain points” in the development of siRNA that severely hamper their clinical application.^[^ ^71^ ^]^ Therefore, targeted delivery technology is the key to siRNA pharmaceutical products. A qualified delivery technology needs to simultaneously ensure accurate delivery, maintain siRNA activity, and be high safety. Dang et al. developed a spherical cationic polypeptide with a stable helical structure, which had a strong membrane‐penetrating capability and could efficiently deliver siPKM2 to tumor cells.^[^ ^72^ ^]^ Similarly, other studies coated siPKM2 with MOFs to protect it against nuclease degradation and realize the reprogramming of glucose metabolism in tumor cells.^[^ ^73^ ^]^ In addition to serving as a carrier, the high reactivity of nanoparticles has also attracted the attention of researchers. Through elaborate structural design, nanoparticles can specifically adsorb and selectively deplete the raw materials for the synthesis of specific proteins. Since intracellular arginine plays a critical role in PKM2 synthesis, specific depletion of arginine in tumor cells via nanosystem can block the synthesis of PKM2. Fang et al. constructed an arginine aptamer‐modified artificial nanozyme (AptCCN) that could specifically capture and oxidize arginine, thus downregulating PKM2.^[^ ^74^ ^]^ Compared with natural enzymes, this artificial nanozyme exhibited higher stability, but there is still much room for improvement in terms of catalytic efficiency and specificity.

**Figure 6:**
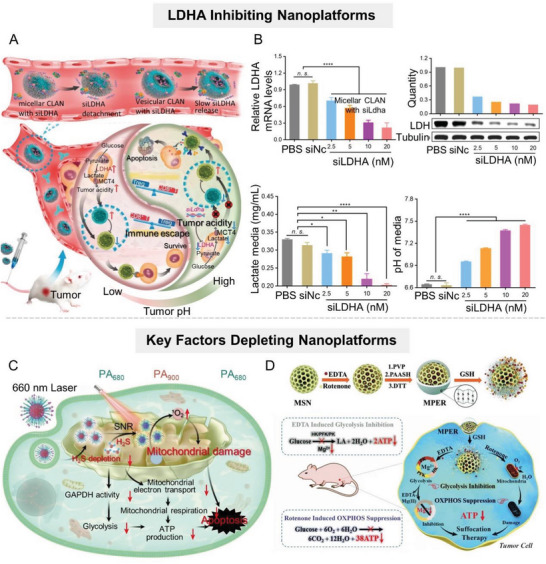
Nanomaterial‐mediated lactate anabolism inhibition by inhibiting glycolysis‐related enzymes LDHA and depleting key factors. A) Scheme of the RNAi nanoparticle‐mediated tumor acidity regulation. B) LDHA downregulation, lactate reduction, and acidity attenuation mediated by RNAi in vitro. Reproduced with permission.^[^ ^78^ ^]^ Copyright 2019, American Chemical Society. C) Scheme diagram of the H_2_S‐depleting nanoplatform to inhibit glycolysis by downregulating GAPDH activity in tumor cells. Reproduced with permission.^[^ ^79^ ^]^ Copyright 2022, Springer Nature. D) Scheme of MSN‐based nanoplatform for codelivering rotenone and Mg^2+^ to downregulate glycolytic‐related enzymes for tumor therapy. Reproduced with permission^[^ ^51^ ^]^ under the terms of the CC BY 3.0 Creative Commons license (https://www.creativecommons.org/licenses/by/3.0/). Copyright 2018, Royal Society of Chemistry.

LDHA, the main subtype of LDH in highly glycolytic tissues, is responsible for converting pyruvate into lactate and is a key enzyme in the final step of glycolysis.^[^ ^75^ ^]^ Numerous studies have demonstrated that the high expression of LDHA is positively associated with tumor development, metastasis, and poor prognosis.^[^ ^75^, ^76^ ^]^ At present, various nanocarriers have been employed to deliver therapeutic agents targeting LDHA.^[^ ^77^ ^]^ For instance, Zhang et al. utilized vesicular cationic lipid‐assisted nanoparticles for delivering siRNA to silence LDHA gene in tumors (**Figure** **6**A).^[^ ^78^ ^]^ The downregulated LDHA expression significantly reduced lactate production and mediated the neutralization of tumor acidity (Figure 6B), which led to the increase of CD8^+^ T/T_reg_ (regulatory T cells) ratio in tumors, holding promise for T cell‐associated immunotherapy.

Apart from the therapeutic targets mentioned above, strategies against other glycolytic proteases including pyruvate dehydrogenase kinase 1 (PDK1) and GAPDH, have also achieved favorable antitumor effects in vivo.^[^ ^79^ ^]^ For example, targeted delivery of PDK1 inhibitors dichloroacetate (DCA) or JX06 by nanosystems has been demonstrated to effectively reduce the PDK1 level and consequently inhibit glycolysis.^[^ ^80^ ^]^

Some nanoparticles were designed to specifically deplete key factors in the glycolysis process, including metal ions and gas signaling molecules, to simultaneously interfere with multiple glycolysis steps.^[^ ^81^ ^]^ It is worth noting that some gaseous signaling molecules, such as NO, H_2_S, and CO, play an important role in the formation and progression of tumors, and affect the glucose metabolism of tumor cells to a certain extent.^[^ ^82^ ^]^ For example, NO and CO can directly inhibit mitochondrial respiration, and thus activate the glycolysis pathway.^[^ ^82^ ^]^ H_2_S, as the third gasotransmitter recently identified, has extensive physiological and pathological effects in the occurrence and development of tumors, especially colorectal tumors.^[^ ^83^ ^]^ Overproduced H_2_S improves the energy supply of tumor cells, enhances glucose uptake, and increases the glycolytic rate and lactate production, thus promoting the survival and metastasis of tumor cells. Given this, Shi's group developed an H_2_S‐depleting nanoplatform, which was proven to reduce GAPDH activity in tumor cells, thereby inhibiting glycolysis (Figure 6C).^[^ ^79^ ^]^ In addition, metal ions are indispensable in many biological processes, and it is estimated that at least one‐third of enzymes in the body require metal ions to function.^[^ ^84^ ^]^ Typically, magnesium ion (Mg^2+^) functions as a key cofactor for glycolytic kinases and plays an important role in the transfer of phosphate groups onto targeted substrates.^[^ ^85^ ^]^ Therefore, specific removal of Mg^2+^ from tumor cells would impair the function of a series of kinases and inhibit glycolysis.^[^ ^86^ ^]^ Shi's group put forward a mesoporous silica nanoparticle (MSN)‐based nanoplatform, denoted as MPER, for codelivery of mitochondrial respiratory inhibitor rotenone and Mg^2+^ chelator ethylenediaminetetraacetic acid (EDTA) into the tumor site (Figure 6D).^[^ ^21^ ^]^ Experiments showed that the expression of the key glycolytic enzymes in tumor cells (including HK2, PFK1, and PKM2) was significantly downregulated after coculture with MPER nanoparticles.

In addition, the inhibitory effect of nanomaterials on glycolytic enzyme activity has also attracted the attention of researchers.^[^ ^87^ ^]^ Xu et al. found that selenium nanoparticles (SeNPs) could suppress glycolysis and lactate production by downregulating the activities of HK2 and pyruvate kinase (PK) in tumor cells.^[^ ^88^ ^]^ Likewise, boric acid (BA) is a classic glycolysis inhibitor, which antagonizes glucose phosphorylation during the first step of glycolysis.^[^ ^89^ ^]^ Based on this, Islam et al. constructed a BA complex composed of styrene–maleic acid copolymer, glucosamine, and BA. The complex could release free BA in the acidic TME to inhibit glycolysis and thereby lead to tumor suppression.^[^ ^90^ ^]^

### 2.2 Promoting Lactate Catabolism

Due to the enhanced glycolysis of tumor cells, the lactate level in tumor tissues is up to 4–40 mM, which is much higher than that in normal tissues.^[^ ^91^ ^]^ Proton‐coupled lactate efflux from tumor cells or stromal cells facilitates the formation of an acidic protumor microenvironment, including promoting proliferation, invasion, angiogenesis, metastasis, drug resistance, immune evasion, etc.^[^ ^6^, ^8^, ^92^ ^]^ Therefore, promoting lactate catabolism in tumor tissues is appealing as a strategy for reversing the protumor microenvironment to improve the tumor therapeutic efficacy (**Table** **3**). At present, a variety of micro/nanosystems have been developed for promoting lactate decomposition, which can be divided into three categories: natural biological enzyme‐based nanosystems, artificial nanozymes, and live bacteria.

**Table 3:** Summary of nanomaterial‐mediated lactate catabolism.

Key nanomaterials	Drugs/therapeutics	Tumor cell types	Refs.
Polymer nanocarriers	LOX	4T1, NSCLC, H1975	[94, 107, 184]
Liposomal nanocarriers	LOX	4T1	[91, 98]
Metal–organic coordination nanocomplex	LOX	4T1, Hepa1–6	[97, 104, 178]
Nanogels	LOX	SMMC‐7721	[100]
Noble mental	LOX	4T1	[101]
Protein nanoparticles	LOX	CT26	[102]
Supramolecular micelles	LOX	B16	[121]
Self‐assembled nanoparticles	LOX	U251	[183]
MSN	LOX	4T1	[99, 106]
MnO_2_	*S. oneidensis* MR‐1, LOX	CT26, B16F10	[117, 120]
nMOF	LOX, *S. oneidensis* MR‐1, yeast	4T1, CT26, HepG2	[94, 97, 113, 116]
SnSe nanozyme	LDH‐like activity	4T1	[87a]
Co*_x_*–N nanocomposite	LOX‐like activity	4T1	[109]

#### 2.2.1 Natural Biological Enzyme‐Based Nanosystems

Natural biological enzymes are biocatalysts that accelerate biochemical reactions in living systems. They are essential for cell proliferation, maintenance, and apoptosis. Due to their remarkable catalytic activity, mild reaction conditions, and high specificity and affinity for target substrates, natural enzymes have advantages over traditional drugs. With the development of biotechnology, natural enzymes have been used in a wide variety of diseases and thus play an important role in the medical field.^[^ ^93^ ^]^ Therefore, using natural enzymes to catalyze lactate consumption in TME is a feasible therapeutic strategy.^[^ ^94^ ^]^ The common natural enzymes that can catalyze lactate consumption include lactate oxidase (LOX) and lactate dehydrogenase B (LDHB).

**Figure 7:**
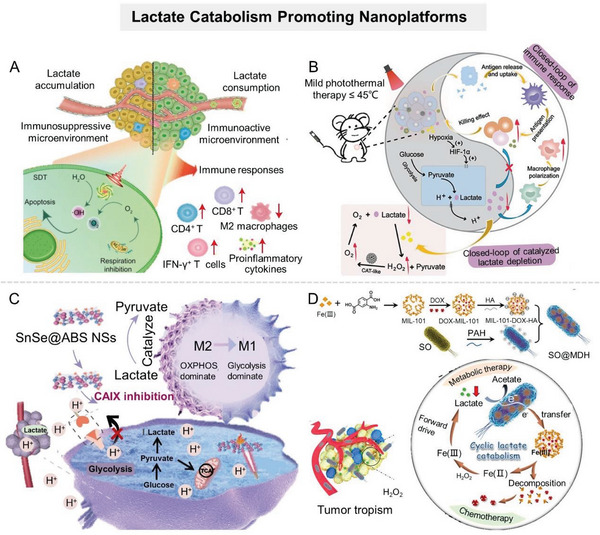
Nanomaterial‐mediated promotion of lactate catabolism. A) Scheme of metal–phenol network encapsulated with LOX and ATO for efficient consumption of lactate. Reproduced with permission.^[^ ^104^ ^]^ Copyright 2021, American Chemical Society. B) Scheme of Cu^2+^‐chelated mesoporous polydopamine (mPDA) nanoparticles encapsulated with LOX to achieve closed‐loop catalysis of lactate consumption in tumor tissues. Reproduced with permission.^[^ ^107^ ^]^ Copyright 2022, American Chemical Society. C) Scheme of SnSe@ABS NSs with LDHB‐like activity to ameliorate the acidic TME. Reproduced with permission.^[^ ^110^ ^]^ Copyright 2022, American Chemical Society. D) Schematic illustration of the bioreactor SO@MDH for active tumor targeting and anaerobical lactate depletion. Reproduced with permission.^[^ ^113^ ^]^ Copyright 2021, American Chemical Society.

LOX is a flavin mononucleotide (FMN)‐dependent enzyme produced by bacteria (such as *Aerococcus viridans*). It catalyzes the conversion of lactate to pyruvate and H_2_O_2_ in the presence of O_2_.^[^ ^95^ ^]^ Notably, LOX, as a flavoprotein, binds tightly to FMN and directly uses O_2_ as the electron transfer substrate without the involvement of additional free coenzymes, promoting the biomedical application of LOX.^[^ ^96^ ^]^ By contrast, catalytic conversion of lactate to pyruvate by LDHB requires NAD^+^ as an electron acceptor, limiting its application. Therefore, the current tumor treatment research focuses primarily on LOX. However, the therapeutic efficacy of LOX is limited by rapid clearance, easy degradation or inactivation, off‐target effects, and toxicity. To improve the stability of LOX and its enrichment in tumor sites, some nanosized carriers, such as MOF,^[^ ^94^, ^97^ ^]^ cationic liposomes,^[^ ^91^, ^98^ ^]^ MSN,^[^ ^99^ ^]^ hydrogel,^[^ ^100^ ^]^ noble metal,^[^ ^101^ ^]^ protein nanoparticles,^[^ ^102^ ^]^ etc., have been developed for tumor‐targeted delivery of LOX. Nevertheless, the catalytic efficiency of LOX is greatly limited by the insufficient supply of O_2_ at tumor sites.^[^ ^103^ ^]^ To address this limitation, Dai's group constructed a nanocomplex (PALF) based on the metal–phenol network that encapsulated LOX and the mitochondrial respiratory inhibitor (atovaquone, ATO) to improve the catalytic efficiency of LOX at the tumor site, efficiently consuming lactate (**Figure** **7**A).^[^ ^104^ ^]^ ATO could significantly reduce mitochondrial oxygen consumption to increase the O_2_ content within tumors, thus promoting LOX‐based lactate consumption. In vitro and in vivo experiments showed that PALF effectively catalyzed lactate depletion, promoted immune cell function recovery, and enhanced antitumor immune responses. Notably, the strategy of alleviating local hypoxia by inhibiting tumor cell respiration is greatly limited by the original oxygen content in tumor tissues, which is extremely low.^[^ ^105^ ^]^ Therefore, some researchers have designed double‐enzyme engineered nanosystems, which could simultaneously deliver LOX and catalase for cascade catalysis of lactate exhaustion.^[^ ^91^, ^106^ ^]^ LOX catalyzed the conversion of lactate to pyruvate and H_2_O_2_ while consuming oxygen, and the by‐product H_2_O_2_ was then decomposed to O_2_ with catalase. These two enzymatic reactions formed a closed loop of lactate consumption. For example, Zheng et al. utilized Cu^2+^‐chelated mesoporous polydopamine (mPDA) nanoparticles encapsulated with LOX (named mCuLP) to achieve closed‐loop catalysis of lactate consumption in tumor tissues (Figure 7B).^[^ ^107^ ^]^ Once mCuLP arrived at the tumor site, Cu^2+^‐chelated mPDA with catalase activity would catalyze the decomposition of H_2_O_2_ to increase the local oxygen level, encourage LOX‐based lactate consumption in the TME, and activate antitumor immunity.

Despite the progress of LOX delivery strategies in tumor therapy, the natural enzyme is readily inactivated and costly, impeding its further development. This initiated the development of artificial nanozymes.

#### 2.2.2 Artificial Nanozymes

Nanozymes are a class of nanomaterials with enzyme‐mimetic activities that can perform biocatalytic reactions of natural enzymes. Compared with natural enzymes, nanozymes have the advantages of facile synthesis, adjustable catalytic activities, robustness to harsh environments, easy modification, and low manufacturing cost, making them widely studied in the biomedical field. As nanotechnology advances, various nanomaterials with unique catalytic properties have broadened the toolbox of artificial enzymes. Currently, nanozymes are primarily composed of metals or metal oxides because the metallic active center can successfully mimic natural enzyme‐catalyzed electron transfer processes.^[^ ^108^ ^]^ To encourage the development of enzyme‐catalyzed lactate conversion in the biomedical field, several nanozymes with catalytic activities similar to LOX or LDHB have been developed, such as SnSe nanosheet,^[^ ^87^ ^]^ NiO@Au nanocomposite,^[^ ^87^ ^]^ Co_4_N/C,^[^ ^109^ ^]^ etc. Significantly, due to their inherent nanomaterial properties, these nanozymes not only serve as a simple alternative to natural enzymes but also provide a multimodal platform for tumor therapy. Ling et al. constructed a nanosystem (SnSe@ABS NSs) to ameliorate the acidic TME by utilizing the SnSe nanosheets with LDH‐like activity loaded with a CAIX inhibitor (benzenesulfonamide, ABS) (Figure 7C).^[^ ^110^ ^]^ SnSe nanosheets could consume lactate to reverse the acidic and immunosuppressive TME, thereby promoting antitumor immune responses. Meanwhile, ABS prevented the conversion of CO_2_ into HCO_3_^−^ and H^+^ to synergistically ameliorate TME acidification. Experimental results verified the capability of SnSe@AS to boost antitumor immunity and prevent tumor metastasis.

In addition, inspired by the nitrogen‐centered structural features of LOX, Zhao et al. constructed LOX‐mimicking Co_4_N/C nanozyme (Co_4_N/C NEs).^[^ ^109^ ^]^ This coordination strategy for mimicking LOX was based on engineering the electronic properties of N centers by adjusting the Co number near N in CO*_X_*–N nanocomposites. Experimental data showed that Co_4_N/C NEs could catalyze the oxidation of lactate to pyruvate at room temperature efficiently in vitro. Moreover, it demonstrated superior lactate catabolism ability in vivo, effectively reversing the lactate‐induced immunosuppression environment by increasing the number of CD8^+^ T cells and decreasing the number of T_reg_ cells. This designing strategy of nanozymes by mimicking natural enzymes provides an important theoretical basis for a deeper understanding of enzyme evolutionary laws and the new generation of artificial nanozyme designs.

Notably, the urgent challenge for scientists is that, despite their superior stability, artificial nanozymes exhibit significantly lower enzymatic activity than most natural enzymes. The route to obtaining low‐cost and highly efficient artificial enzymes awaits further exploration.

#### 2.2.3 Live Bacteria

In addition, some naturally occurring microorganisms present the ability to metabolize lactate.^[^ ^111^ ^]^ Some bacteria can use lactate as a metabolic substrate, such as *Gluconobacter oxydans*,^[^ ^112^ ^]^ *Shewanella oneidensis*,^[^ ^113^ ^]^ *Eubacterium hallii*,^[^ ^114^ ^]^ *Anaerostipes spp*.,^[^ ^114^ ^]^ and *veillonella atypica*.^[^ ^115^ ^]^ Live bacteria have better stability than natural enzymes and higher catalytic efficiency than artificial nanozymes in vivo. Importantly, certain bacteria hold an inherent tumor tropism and can actively penetrate deep into tumors, allowing them to enrich at tumor sites and enhance antitumor effects.^[^ ^111^, ^116^ ^]^ Typically, *Shewanella oneidensis* MR‐1 (*S. oneidensis* MR‐1), a widespread extracellular respiratory bacterium in the environment, can utilize lactate as an energy source for respiration. In this process, *S. oneidensis* MR‐1 mediates the transfer of electrons between lactate and metallic minerals.^[^ ^117^ ^]^ Based on this, Zhang's group integrated *S. oneidensis* MR‐1 with DOX‐loaded MIL‐101 nanoparticles to fabricate an intelligent bioreactor (SO@MDH) that could actively target and colonize hypoxic tumor regions while anaerobically depleting lactate (Figure 7D).^[^ ^113^ ^]^ In this bioreactor, MIL‐101 nanoparticles interacted closely with *S. oneidensis* MR‐1. *S. oneidensis* MR‐1 catalyzed the oxidation of lactate and transferred electrons to the Fe^3+^ in MIL‐101 nanoparticles, causing the collapse of the MIL‐101 nanostructure to release chemotherapeutic DOX. The experimental results showed that SO@MDH could downregulate the expression of multidrug resistance‐related proteins through the decomposition of lactate, and significantly improve the efficacy of chemotherapy. In addition, one intestinal bacterium, *veillonella atypica*, isolated from stool samples of marathon runners in recent years, can break down the lactate accumulated in the intestine of the host during exercise.^[^ ^118^ ^]^ This ability of *veillonella atypica* to decompose lactate makes it a potential candidate for the treatment of colorectal tumors. It is important to note that the potential risks must be considered when using live microorganisms for disease treatment. Although current advances in synthetic biology allow for the attenuation and elimination of therapeutic bacteria's biotoxicity, the risk of bacteria regaining pathogenicity cannot be fully excluded due to their great potential for mutation. Therefore, living bacteria still need to undergo a long‐term and rigorous validation process before being extended to clinical applications. Additionlly, some cell‐derived bioactive components are also expected to inspire the construction of tumor‐targeting nanoregulator.^[^ ^119^ ^]^

**Figure 8:**
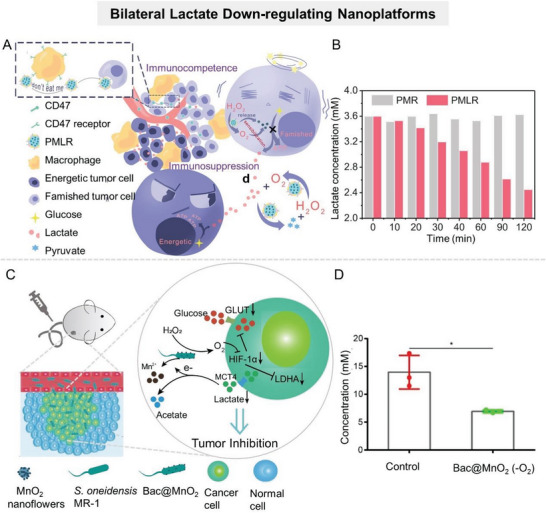
Nanomaterial‐mediated lactate production inhibiting and depletion promoting. A) Schematic illustration of the cascade catalytic nanosystem (PMLR) to efficiently deplete intra/extracellular lactate in TME. B) Lactate concentration in solution of PMR and PMLR nanosystems. Reproduced with permission.^[^ ^120^ ^]^ Copyright 2019, Wiley‐VCH. C) Schematic diagram of the antitumor mechanism of Bac@MnO_2_. D) Bac@MnO_2_ mediated lactate concentration in CT26 tumor tissues. Reproduced with permission.^[^ ^117a^ ^]^ Copyright 2020, Wiley‐VCH.

Although promoting lactate decomposition reverses the protumor microenvironment to some extent, the therapeutic efficacy of this strategy is partially offset by rapid and continuous lactate production in tumors with enhanced glycolysis. Therefore, the effect of unilateral consumption of lactate on tumor energy metabolism and TME regulation is limited. It is expected to achieve more effective lactate metabolic regulation via constructing nanosystems that function “bilaterally” to reduce the lactate pool of TME, that is, to suppress lactate anabolism while motivating lactate catabolism. To this end, Zhang's group constructed a cascade catalytic nanosystem (PMLR) to efficiently deplete lactate in TME (**Figure** **8**A).^[^ ^120^ ^]^ PMLR was prepared by loading LOX and glycolysis inhibitor 3‐­(3‐­pyridinyl)‐­1‐­(4­pyridinyl)­‐2‐­propen‐­1‐­one (3PO) into hollow MnO_2_ that was further camouflaged with erythrocyte membrane (mRBC). The mRBC camouflage allowed the nanosystem to have a longer circulation time in vivo, which was conducive to its enrichment in tumor sites. Extracellularly, LOX within PMLR nanosystem catalyzed lactate oxidation, thus depleting lactate in the TME. Meanwhile, within tumor cells, the PMLR nanosystem released glycolysis inhibitor 3PO to block glycolysis, inhibiting lactate production (Figure 8B). In addition, MnO_2_ in the PMLR nanosystem catalyzed H_2_O_2_ decomposition to produce O_2_, which could sensitize both extracellular and intracellular processes. The experimental results showed that the PMLR nanosystem could efficiently deplete lactate and inhibit ATP production in highly glycolytic tumors, and awaken antitumor immunity. Similarly, Zhang's group constructed a biohybrid micro/nanosystem (defined as Bac@MnO_2_) by integrating MnO_2_ nanoflowers with *S. oneidensis* MR‐1 to simultaneously decompose lactate and inhibit its production (Figure 8C).^[^ ^117^ ^]^ Lactate, bacteria, and MnO_2_ formed a complete respiratory pathway in the tumor tissues, leading to the continuous decomposition of lactate in TME (Figure 8D). Meanwhile, MnO_2_ nanoflowers could downregulate the expression of HIF‐1α by catalyzing O_2_ generation, suppressing lactate anabolism. In addition, some researchers have constructed bioreactors to consume lactate and its raw materials (glucose) at the same time.^[^ ^121^ ^]^ For example, Wang et al. integrated *Saccharomyces cerevisiae* capable of metabolizing glucose with LOX‐encapsulated MOF to synergistically reduce lactate levels at tumor sites.^[^ ^116^ ^]^

Various strategies have been developed to promote lactate decomposition at tumor sites, but their antitumor efficacy is compromised by the complex and dynamic microenvironment of tumor sites. To improve the efficiency of lactate decomposition by nanosystems, researchers should also consider the biological barriers at tumor sites, such as hypoxia, high interstitial pressure, and overexpression of glycolytic‐related enzymes.

### 2.3 Disrupting “Lactate Shuttle”

**Figure 9:**
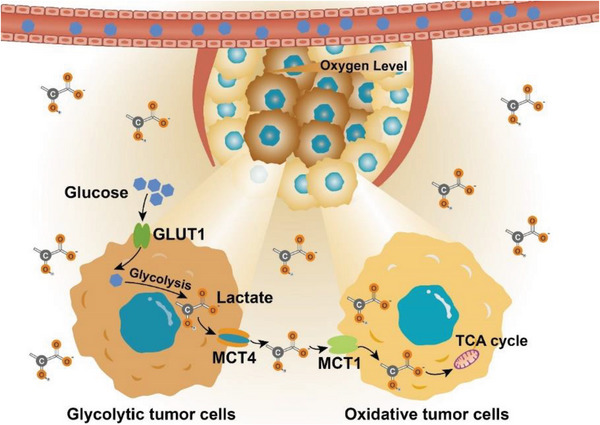
Lactate shuttles between tumor cells. Tumor cells far from the vessels opt for glycolysis instead of OXPHOS to provide a vast amount of energy for rapid proliferation due to the shortage of oxygen, increasing lactate production. Lactate is then discharged into the extracellular environment through monocarboxylic acid transporter 4 (MCT4), ultimately acidifying the TME. The catabolites lactate is in turn acquired by tumor cells closer to the vessels through monocarboxylic acid transporter 1 (MCT1), then entered into the tricarboxylic acid (TCA) cycle to fuel mitochondrial metabolism for energy generation.

The abnormally high expression of monocarboxylate transporters (MCTs) on tumor cell membranes can mediate the rapid transmembrane transport of lactate, known as the “lactate shuttle,” which promotes the crosstalk between tumor cells and the microenvironment.^[^ ^6^, ^8^ ^]^ In solid tumors, the interior hypoxic tumor cells produce large amounts of lactate via accelerated glycolysis, which is then excreted into the extracellular microenvironment via MCT4 highly expressed in hypoxic tumor cells.^[^ ^8^ ^]^ Subsequently, peripheral oxygen‐rich tumor cells overexpressing MCT1 take up lactate and converted it to pyruvate, which serves as fuel for respiration (**Figure** **9**).^[^ ^122^ ^]^ This metabolic heterogeneity, caused by the difference in oxygen and nutrient supply, guarantees an effective energy supply to tumor cells in different ecological niches under adverse conditions, which is conducive to the metabolic symbiosis between tumor cells.^[^ ^6^, ^123^ ^]^ Published studies have shown that many tumors overexpress MCTs. For example, the mRNA and protein expression levels of MCT4 in cervical carcinoma (HeLa) cells were upregulated under hypoxic conditions.^[^ ^124^ ^]^ Some tumors, including glioblastoma, colon tumor, and breast cancer, simultaneously upregulate the mRNA and protein expression levels of MCT1 and MCT4, promoting tumor progression, chemoresistance, and immune suppression.^[^ ^125^ ^]^ Therefore, interfering with metabolic symbiosis by disrupting the “lactate shuttle” is a promising strategy for tumor therapy, i.e., blocking the flow of lactate between tumor cells by inhibiting MCT4‐mediated lactate efflux of hypoxic tumor cells or MCT1‐mediated lactate influx of oxygen‐rich tumor cells, thus destroying the energy competitive advantage of tumor cells under adverse conditions.^[^ ^57^, ^126^ ^]^ In recent years, a series of nanoformulations targeting “lactate shuttle” have been reported, all of which have demonstrated exceptional antitumor activity (**Table** **4**).

**Table 4:** Summary of nanomaterial‐mediated “lactate shuttle” disruption.

Mechanism	Key nanomaterials	Targets	Drugs/therapeutics	Tumor types	Refs.
Lactate efflux inhibition	Polymer nanocarriers	MCT4	Syrosingopine, LND	4T1, B16F10	[130, 208]
	Silicon‐based nanoparticles	MCT4	siMCT4	B16F10, 4T1	[132]
	Prussian blue	MCT4	siMCT4	4T1, Huh‐7	[129c]
	CaP	MCT4	siMCT4	MCF7	[129a]
	Iron oxide	MCT4	siMCT4	PC3	[129b]
	Self‐assembled nanodrug	MCT4	LND	CT26	[131]
	FeS	–	H_2_S	4T1	[139a]
	CuSe/CoSe_2_ nanocomposites	MCT4	Syrosingopine	4T1	[209]
	Fe_3_O_4_ nanoparticles	MCT4	Syrosingopine	B16F10	[130d]
	nMOFs	MCT4	Syrosingopine	Hepa1–6	[97c]
Lactate influx inhibition	nMOFs	MCT1	α‐CHC	CT26, MCF‐7	[144, 146]
	Self‐assembled nanodrug	MCT1	α‐CHC	CT26	[210]
“Lactate shuttle” inhibition and anabolism interference	Polymer nanocarriers	MCT4	α‐CHC and l‐Cys^a)^	MCF‐7	[133b]
	Silicon‐based nanoparticles	MCT4 and TCA cycle	Flu and Me	4T1, MCF‐7	[133a,c]
	Liposomal nanocarriers	MCT4 and HK	Syrosingopine and LND	4T1	[166]

^a)^ A H_2_S donor.

#### 2.3.1 Inhibiting Lactate Efflux

**Figure 10:**
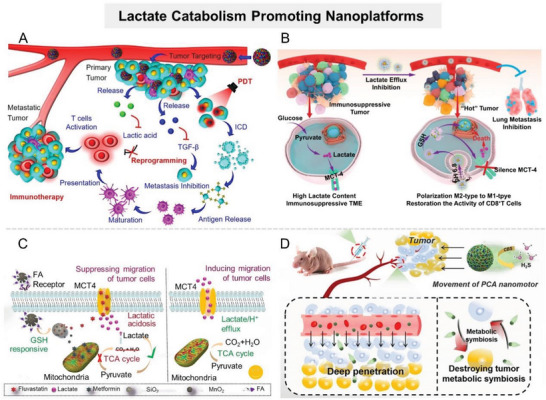
Nanomaterial‐mediated disruption of lactate efflux. A) cheme of TerBio self‐assembled by Ce6, Lon, and SB for lactate efflux inhibition and enhanced tumor treatment. Reproduced with permission.^[^ ^131^ ^]^ Copyright 2022, American Chemical Society. B) The antitumor mechanism of the cascaded responsive nanoplatform HMONs@HCPT–BSA–PEI–CDM–PEG@siMCT4. Reproduced with permission.^[^ ^132^ ^]^ Copyright 2020, American Chemical Society. C) Scheme of Me&Flu@MSN@MnO_2_‐FA interfering with lactate metabolism to induce intracellular acidosis to suppress metastasis. Reproduced with permission.^[^ ^133c^ ^]^ Copyright 2020, Royal Society of Chemistry. D) Schematic illustration of PCA nanomotor for tumor metabolism symbiosis disruption. Reproduced with permission.^[^ ^133b^ ^]^ Copyright 2021, Wiley‐VCH.

Highly glycolytic tumor cells continuously produce lactate, leading to rapid intracellular acidification, which reduces cell viability and inhibits glycolytic enzyme activities like PFK1.^[^ ^127^ ^]^ To maintain glycolytic rate and cell viability, glycolytic tumor cells continuously export lactate via the overexpressed MCT4.^[^ ^6^, ^8^, ^128^ ^]^ Therefore, it is a feasible strategy for improving tumor treatment outcomes via downregulating the expression or activity of MCT4 to inhibit lactate leakage, which results in glycolytic tumor cell acidosis or the destruction of metabolic symbiosis between tumor cells.^[^ ^129^ ^]^ Currently, some MCT4 small‐molecule inhibitors have been developed, such as LND, pyrazole derivatives, and syrosingopine^[^ ^130^ ^]^ (Table 2). Among them, LND, a derivative of indole‐3‐carboxylic acid, is capable of inhibiting glycolysis and energy metabolism of tumor cells and has demonstrated safety in phase II and phase III clinical trials. However, its clinical efficacy remains limited due to nonselective distribution in vivo.^[^ ^130^ ^]^ To optimize pharmacokinetic properties and achieve targeted delivery of LND, Zhao et al. developed a carrier‐free nanodrug delivery system (denoted as TerBio) that was self‐assembled from Ce6, SB505124 (SB), and LND (**Figure** **10**A).^[^ ^131^ ^]^ Ce6‐mediated PDT impaired tumor cell membranes, promoting cellular internalization, lysosomal escape, and tumor penetration of TerBio. Therefore, this nanosystem improved the availability of LND at the target site and effectively inhibited lactate efflux from tumor cells, thereby reversing the lactate‐related protumor microenvironment. However, it should be noted that the drug release of such nanosystems assembled by hydrophobic or π–π stacking interactions is often uncontrollable, which fails to effectively balance the safety and therapeutic performance of agents. To achieve controlled drug release, Li et al. constructed an intelligently responsive nanoplatform (HMONs@HCPT–BSA–PEI–CDM–PEG) that provided therapeutic agents with favorable stability, safety, and targeting ability through stepwise responses to the acidic TME and intracellular REDOX (GSH) (Figure 10B).^[^ ^132^ ^]^ Briefly, GSH‐responsive MSN nanoparticles loaded with chemotherapy agents were modified with siMCT4‐loaded polyether imide (PEI) and acid‐detachable PEG fragments. The acid‐detachable PEG segment improved the nanosystem's stability during blood transport while also endowing it with charge reversal capability in response to the TME to promote tumor cell uptake. Within tumor cells, the nanoplatform disintegrated in response to the high level of GSH, releasing chemotherapeutic drugs and siMCT4 to inhibit tumor growth and reverse the immunosuppressive TME.

In addition to improving MCT4 inhibitors distribution at tumor sites, nanosystems have been developed to synergistically interfere with tumor cell lactate metabolism, i.e., blocking lactate efflux while promoting lactate production, thus accelerating lactate accumulation in tumor cells and efficiently inducing tumor cell acidosis.^[^ ^133^ ^]^ For example, Zhang's group selected MSN@MnO_2_ core–shell nanoparticles as carriers to simultaneously deliver mitochondrial respiratory inhibitor metformin (Me) and MCT4 inhibitor fluvastatin sodium (Flu) (Figure 10C).^[^ ^133^ ^]^ As a gatekeeper, the MnO_2_ shell degraded in response to the overexpressed GSH in TME, leading to Me and Flu release. The released Me hindered the aerobic respiration of pyruvate, resulting in increased lactate production in tumor cells. At the same time, the Flu‐induced MCT4 downregulation resulted in limited lactate efflux, synergistically promoting intracellular acidosis. This kind of nanosystem with a dual‐promoting effect on lactate accumulation in tumor cells can not only induce tumor cell apoptosis but also effectively mediate the alteration of the intracellular microenvironment to enhance other therapies. Typically, Jiang et al. loaded Me and Flu in ClO_2_@CaSiO_3_@MnO_2_ to reduce intracellular pH, promoting strongly oxidizing ClO_2_ release from the nanosystem within tumor cells.^[^ ^133^ ^]^

Notably, certain nanosystems are capable of directly interfering with lactate efflux in glycolytic tumor cells by initiating specific chemical reactions at tumor sites to produce energy metabolism‐regulatory factors. For instance, hydrogen sulfide (H_2_S) is an important signaling molecule that appears in various tissues and has significant impacts on several physiological functions, including the regulation of vasodilation and neurotransmitter.^[^ ^134^ ^]^ Recently, published studies have shown that regulating H_2_S concentrations in tumors has great potential to improve tumor therapy outcomes.^[^ ^79^, ^135^ ^]^ Exogenous introduction of H_2_S can promote glucose uptake, aggravate tumor hypoxia, and inhibit proton efflux, thus disrupting tumor cell homeostasis.^[^ ^136^ ^]^ Conventional H_2_S donors, such as GYY4137 and ATB‐346, can slowly and spontaneously release H_2_S in physiological environments. However, their uncontrolled H_2_S production process would result in side effects and unsatisfactory treatment outcomes.^[^ ^137^ ^]^ To achieve controlled and tumor‐site‐specific H_2_S release, some nanosystems have been developed that promote in situ synthesis of H_2_S at tumor sites.^[^ ^138^ ^]^ The most common paradigm is the tumor‐targeting l‐cysteine (l‐Cys) delivery nanosystem, in which the loaded l‐Cys is converted to H_2_S, which is catalyzed by the highly expressed cysteine synthase (CBS) at the tumor site. For example, Wan et al. developed an H_2_S‐driven nanomotor to disrupt tumor metabolic symbiosis based on the poly(sulfobetaine methacrylate) zwitterionic nanoparticles loaded with l‐Cys and α‐cyano‐4‐hydroxycinnamic acid (α‐CHC) (Figure 10D).^[^ ^133^ ^]^ This l‐Cys‐loaded nanomotor produced H_2_S when triggered by CBS in the TME, which facilitated glucose uptake by tumor cells to produce a large amount of lactate and inhibited intracellular protons efflux, ultimately leading to malignant cell acidosis. Besides, α‐CHC inhibited MCT‐1/4 expressions and disrupted the lactate transmission chain between tumor cells, thus aggravating the acidosis process. Of note, some metal sulfides, such as FeS and CuS, also present the ability to produce H_2_S in the acidic TME, promoting the acidosis of tumor cells and metal ion‐mediated chemodynamic therapy (CDT).^[^ ^139^ ^]^

Although inhibition of MCT4 is a promising strategy for antitumor treatment, it should be noted that MCT4 is usually expressed in the hypoxic regions far from blood vessels (deep in tumor tissues), making common nanotherapeutic agents difficult to reach. Therefore, it is expected to promote the efficacy of MCT4 inhibition therapy by elaborately designing nanosystem structures to facilitate MCT4 inhibitor deep penetration into tumors.

#### 2.3.2 Lactate Influx Inhibition

**Figure 11:**
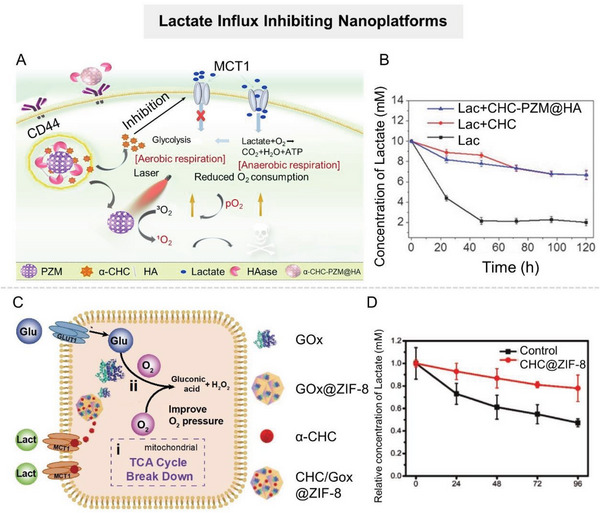
Nanomaterial‐mediated disruption of lactate influx. A) Schematic illustration of the porphyrinic MOF nanoplatform for interfering with the lactate uptake to enhance tumor treatment. B) Lactate concentration of cells after different treatments. Reproduced with permission.^[^ ^144^ ^]^ Copyright 2018, Wiley‐VCH. C) Schematic illustration of the tumor therapeutic mechanisms of CHC/GOX@ZIF‐8. D) Lactate consumption profile of cells in different groups. Reproduced with permission.^[^ ^146^ ^]^ Copyright 2021, Wiley‐VCH.

To coordinate and symbiosis with interior hypoxic tumor cells, peripheral oxygen‐rich tumor cells actively upregulate MCT1 expression to intake high amounts of lactate instead of glucose to fuel OXPHOS.^[^ ^123^, ^140^ ^]^ Basic studies have shown that the high expression of MCT1 is highly correlated with the poor prognosis of tumor patients.^[^ ^140^, ^141^ ^]^ Therefore, MCT1 suppression would disrupt lactate‐fueled aerobic respiration, causing the metabolism patterns of oxygen‐rich tumor cells to shift from OXPHOS to glycolysis.^[^ ^142^ ^]^ This metabolic transformation leads to increased glucose consumption and even glucose deficiency, which hampers glucose uptake by the internal hypoxic tumor cells, ultimately constraining energy metabolism and inducing apoptosis.^[^ ^123^, ^142^ ^]^ Researchers have successfully developed several MCT1 inhibitors, including α‐CHC and its analogues, pyridazinone analogues (e.g., AZD3965, AR‐C155858), coumarin and its analogues (e.g., *N*,*N*‐dialkyl carboxy coumarins)^[^ ^123^, ^126^, ^143^ ^]^ (Table 2). To improve the therapeutic effect of MCT1 inhibitors, Zhang's group incorporated α‐CHC into a porous Zr(IV)‐based porphyrinic MOF (PZM) nanoparticle that was further coated with the tumor‐targeting ligand HA to obtain CHC‐PZM@HA for blocking lactate‐fueled respiration and enhancing PDT (**Figure** **11**A).^[^ ^144^ ^]^ The experimental results demonstrated that CHC‐PZM@HA reduced the expression of MCT1 in tumor cells and weakened lactate uptake, thus disrupting the metabolic balance within the tumor (Figure 11B). This strategy, which alleviated tumor hypoxia by interfering with tumor lactate metabolism rather than carrying or generating oxygen through nanomaterials, provides a new idea for enhancing oxygen‐dependent therapies.

To further improve the inhibitory effect on tumor growth, researchers attempted to construct nanosystems with multiple energy supply blockades that simultaneously inhibit tumor cells from uptaking lactate and other energy sources (e.g., glucose, amino acids, and fatty acids).^[^ ^145^ ^]^ Typically, Yu et al. constructed a nanosystem (CHC/GOX@ZIF‐8) with dual metabolic inhibition by coloading α‐CHC and GOX with zeolitic imidazolate framework‐8 (ZIF‐8) (Figure 11C).^[^ ^146^ ^]^ This nanosystem simultaneously deprived tumor cells of lactate and glucose, the two main fuels for energy metabolism (Figure 11D). In addition, α‐CHC‐mediated metabolism transformation alleviated tumor hypoxia, which enhanced the catalysis activity of GOX. This strategy has been demonstrated to have an excellent tumor suppressive effect by depriving tumor cells of multiple fuels supporting energy metabolism.

Compared with MCT4, which is mainly overexpressed by tumor cells located in the center of solid tumors, MCT1 appears to be a more accessible pharmacological target since it generally occurs in the tumor cells adjacent to blood vessels.^[^ ^123^, ^140^, ^147^ ^]^ Unfortunately, the antitumor efficacy of MCT1‐targeting therapies is often limited, particularly in tumors with large volumes and high heterogeneity. Therefore, the combination of metabolic symbiosis disruption and other therapeutic modalities is a potentially effective antitumor therapy.

## 3 Combining Lactate Metabolic Regulation with Other Therapies

**Figure 12:**
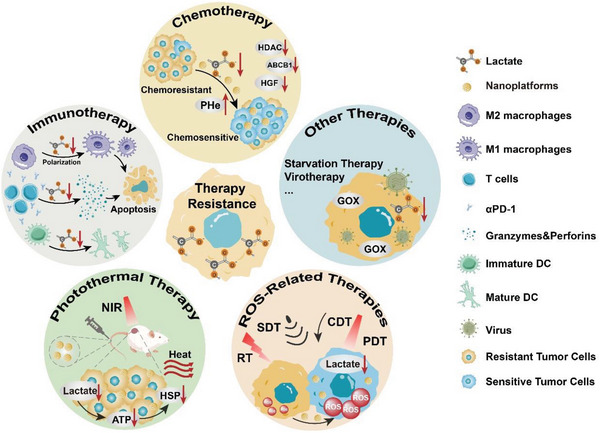
Strategies that combine lactate metabolic modulation with other therapies, including chemotherapy, immunotherapy, photothermal therapy (PTT), ROS‐related therapies, and others.

One of the most important and pressing issues in tumor management is therapy resistance. Lactate accumulated in TME affects the behavior of tumor cells and stromal cells in various aspects and participates in the construction of the continuously changing TME. Numerous studies have shown that lactate greatly drives the development of tumor therapy resistance, and is highly correlated with poor clinical tumor treatment outcomes. Therefore, combining lactate metabolic regulation with other therapies has great potential for achieving synergistic effects and improved antitumor therapeutic outcomes (**Figure** **12**).

### 3.1 Combining Lactate Metabolic Regulation with Chemotherapy

Chemotherapy, as a systemic therapeutic pattern, plays a dominant role in tumor treatment. Chemotherapy resistance is predisposed to tumor recurrence and metastasis, so 80–90% of tumor patient deaths are directly or indirectly attributable to drug resistance.^[^ ^148^ ^]^ With the more intensive investigation into TME and tumor metabolism, numerous studies have confirmed that lactate played a significant role in the occurrence and maintenance of tumor chemoresistance.^[^ ^149^ ^]^

**Figure 13:**
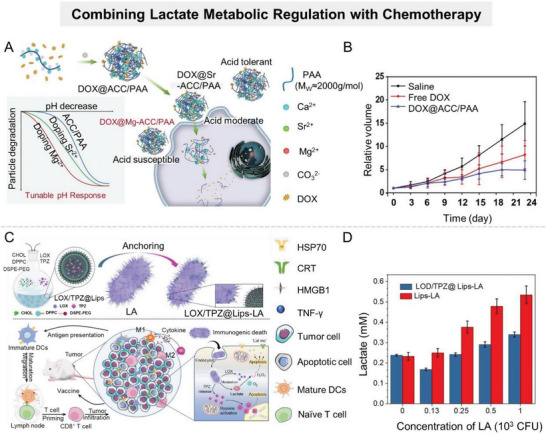
Nanomaterial‐mediated therapy strategies combined lactate metabolic regulation with chemotherapies. A) Schematic diagram of the construction of DOX‐loaded ACC/PAA nanoparticles and their regulation mechanism on tumor cells. Different amounts of Sr^2+^ or Mg^2+^ doped in ACC lead to different degradation curves of nanoparticles, indicating that the pH response of drug release is adjustable. B) Relative tumor volumes with different treatments (*n* = 5). Reproduced with permission.^[^ ^155^ ^]^ Copyright 2019, Wiley‐VCH. C) Scheme of the fabrication of LOX/TPZ@Lips‐LA and its mediated combined therapy. D) Intracellular lactate concentration after different treatments for 12 h. Reproduced with permission.^[^ ^98^ ^]^ Copyright 2020, Wiley‐VCH.

One of the leading mechanisms by which lactate accumulated in tumors mediates chemotherapy resistance is by affecting chemotherapeutic drug uptake and efflux.^[^ ^149^ ^]^ In contrast to normal cells with an intracellular pH (pHi) of ≈7.2 and an extracellular pH (pHe) of ≈7.4, tumor cells continuously produce and export large amounts of lactate, leading to the change of the pH gradient inside and outside the tumor cells, namely, intracellular alkalization (pHi ≈ 7.6) and extracellular acidification (pHe ≈6.7–7.1).^[^ ^150^ ^]^ The acidic TME protonates and charges weakly alkaline chemotherapeutic agents (e.g., paclitaxel, DOX, and mitoxantrone), reducing their cellular permeability and making it difficult for them to enter tumor cells via passive diffusion.^[^ ^151^ ^]^ Furthermore, intracellular alkalization favors tumor cell DNA repair while weakening the binding ability of antitumor drugs such as mitoxantrone and DOX to DNA, which directly induce multidrug resistance.^[^ ^152^ ^]^ Therefore, it is expected to overcome chemoresistance and improve clinical efficacy by changing the distribution of intracellular and extracellular protons to adjust the pH gradient. At present, pH regulators in clinical trials, including carbonic anhydrase inhibitors, proton pump inhibitors, MCT inhibitors, etc., have demonstrated tremendous potential in sensitizing chemotherapy.^[^ ^153^ ^]^ Therefore, developing tumor‐targeted pH regulation strategies is a promising avenue for improving chemotherapy efficacy. Some studies have exploited nanocarriers with excellent tumor‐targeting ability to deliver pH regulators for sensitizing chemotherapy.^[^ ^132^ ^]^ Notably, in addition to serving as targeted drug delivery vehicles, some alkaline nanoparticles, including CaCO_3_, MnO_2_, Ca_3_(PO_4_)_2_, etc., can directly regulate pH in tumors owing to their intrinsic acid‐scavenging ability.^[^ ^154^ ^]^ These alkaline nanoparticles have been shown to selectively accumulate and dissolve at tumor sites, increasing pHe asymptotically to 7.4.^[^ ^154^ ^]^ Among them, CaCO_3_ has received the greatest attention. To further improve the responsiveness of CaCO_3_ nanoparticles to TME, some researchers have modified them. For example, Xu et al. developed an amorphous CaCO_3_/poly(acrylic acid) nanohybrid (ACC/PAA) doped with proper Sr^2+^ and Mg^2+^, which completely degraded in response to slightly acidic TME (**Figure** **13**A).^[^ ^155^ ^]^ Studies showed that this CaCO_3_‐based nanohybrid had a high DOX loading capacity and promoted DOX entry into cells by ameliorating the acidic TME to improve the tumor‐killing effect (Figure 13B).

Apart from affecting drug transport by regulating intracellular/extracellular pH, tumor‐derived lactate can also induce chemotherapy resistance by promoting the expression and secretion of tolerance‐related proteins and cytokines.^[^ ^149^, ^156^ ^]^ For instance, lactate can enhance the DNA repair capability of cervical tumor cells through upregulating histone deacetylase, allowing tumor cells to develop resistance to cisplatin.^[^ ^157^ ^]^ Wagner and co‐workers reported that tumor‐derived lactate upregulated the expression of multidrug resistance protein 1 in HeLa cells to promote DOX efflux, leading to resistance.^[^ ^158^ ^]^ In nonsmall cell lung cancer (NSCLC), lactate induces the secretion of hepatocyte growth factor by tumor‐associated fibroblasts, thus promoting tumor progression and resistance to tyrosine kinase inhibitors.^[^ ^159^ ^]^ Notably, chemotherapeutic drugs can induce the compensatory metabolic of tumor cells to upregulate glycolysis. For example, anthracyclines, taxanes, and platinum have been reported to upregulate the expression of GLUT1, LDHA, PKM2, and PDK3 to promote lactate production, resulting in chemotherapy resistance.^[^ ^160^ ^]^ Therefore, to accomplish efficient chemotherapy, a series of integrated nanoplatforms have been developed to concurrently decrease lactate levels in TME and deliver chemotherapeutic agents.^[^ ^21^, ^25^, ^27^, ^32^, ^41^, ^57^, ^59^, ^113^, ^161^ ^]^ For example, Luo et al. constructed Hb‐LOX‐DOX‐ZIF8@platelet membrane nanosystem (HLDZ@PM NPs) using nano‐ZIF‐8 as the carrier.^[^ ^162^ ^]^ LOX adequately depleted intratumoral lactate, making tumor cells more sensitive to DOX‐induced chemotherapy.

Furthermore, promoting lactate oxidation can also exacerbate hypoxia in tumors, enhancing the therapeutic effect of hypoxic‐activated prodrugs, such as tirapazamine (TPZ) and banoxantrone dihydrochloride.^[^ ^98^, ^99^ ^]^ Typically, Shi's group developed a microbiotic nanomedicine (LOX/TPZ@Lips‐LA) by anchoring liposomes loaded with LOX and TPZ onto the surface of lactobacillus (LA) (Figure 13C).^[^ ^98^ ^]^ LA endowed nanomedicine with tumor‐targeting properties, enabling it to effectively deliver TPZ and LOX to tumor tissues. When LOX/TPZ@Lips‐LA reached tumor sites, LOX catalyzed lactate oxidation by LA metabolism to sufficiently consume intratumoral oxygen, which activated the low‐chemo toxic prodrug TPZ to induce tumor cell apoptosis (Figure 13D).

Despite the great potential of intervening in tumor lactate metabolism to sensitize chemotherapy,^[^ ^149a^ ^]^ it is still necessary to explore the optimal scheme of organically combining lactate metabolic regulation with chemotherapy, such as screening the most effective lactate regulation target, the best treatment time window and the most appropriate action site (intracellular or extracellular) to maximize the antitumor efficacy.

### 3.2 Combining Lactate Metabolic Regulation with Immunotherapy

Immunotherapy, which activates the body's immune system to fight against tumors, has shown broad prospects in preventing tumor metastasis and recurrence in recent years.^[^ ^163^ ^]^ Nevertheless, most tumor patients do not respond well to immunotherapy, mainly because tumors develop multiple defense mechanisms against antitumor immunity. Notably, lactate enriched in the TME has been demonstrated to promote the formation of detrimental immunosuppressive networks, thereby crippling antitumor immunity. The main events underlying this promotion are as follows. 1) Lactate promotes the differentiation of IL‐10‐producing tolerogenic dendritic cells (DC‐10) to suppress the antigen‐presenting ability of DCs. 2) Lactate attenuates the activities of effector cells (e.g., natural killer (NK) cells and cytotoxic T cells (CTLs)) by inhibiting the secretion of tumor‐killing cytokines such as perforins, granzymes, and IFN‐γ, impairing their immune‐killing effect. 3) Lactate promotes the differentiation and recruitment of immunosuppressive cells, such as T_reg_ cells and myeloid‐derived suppressor cells (MDSCs), assisting tumor cell immune escape.^[^ ^6^, ^10^ ^]^ These collectively reveal the critical role of lactate in tumors establishing resistance to immunotherapy and imply that tumor immunotherapy would benefit from lactate metabolic regulation.

**Figure 14:**
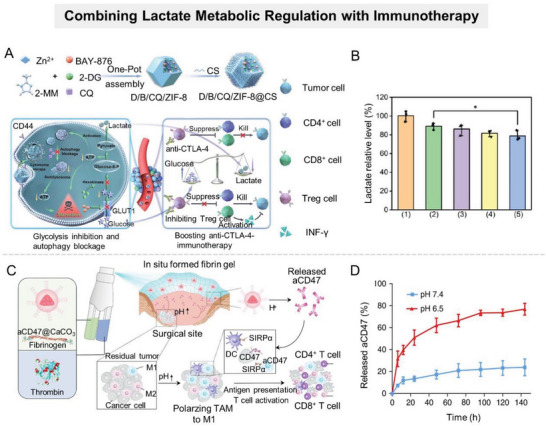
A) Schematic diagram of the construction of D/B/CQ/ZIF‐8@CS and its synergetic mechanism for starvation therapy and lactate metabolic regulation. B) Extracellular lactate level of 4T1 cells after various treatments for 24 h. (1) PBS, (2) B@ZIF‐8@CS, (3) D@ZIF‐8@CS, (4) D/B@ZIF‐8@CS, and (5) D/B/CQ@ZIF‐8@CS. Reproduced with permission.^[^ ^165^ ^]^ Copyright 2022, American Chemical Society. C) Scheme of the fibrin gel containing aCD47@CaCO_3_ nanoparticles for the combination of H^+^ neutralization and immune system awakening. D) aCD47 release profiles from fibrin in solutions at pH 6.5 and 7.4. Reproduced with permission.^[^ ^164^ ^]^ Copyright 2018, Springer Nature.

Recently, several strategies have been developed to sensitize antitumor immunotherapy by reversing the immunosuppressive TME caused by lactate accumulation, including the inhibition of glycolysis to reduce lactate production,^[^ ^20^, ^21^, ^80^ ^]^ depletion of intratumoral lactate by LOX,^[^ ^106^ ^]^ blockage of lactate efflux to the extracellular microenvironment^[^ ^132^ ^]^ or direct neutralization of excess H^+^.^[^ ^164^ ^]^ For example, a metabolic nanoregulator (D/B/CQ@ZIF‐8@CS) encapsulating antiglycolytic agent 2‐DG, GLUT1 inhibitor BAY‐876, and autophagy inhibitor chloroquine (CQ) was developed for synergetic tumor metabolic regulation‐immunotherapy (**Figure** **14**A).^[^ ^165^ ^]^ The combination of BAY‐876 and 2‐DG cut off the lactate production from the source through dual inhibition of glucose uptake and glycolysis process in tumor cells, thus effectively reversing the lactate‐related immunosuppressive TME to augment anti‐CTLA‐4‐based immunotherapy (Figure 14B). In another study, a nano‐prodrug assembled by a dimer composed of glycolysis inhibitor LND and indoleamine 2,3‐dioxygenase‐1 inhibitor NLG919 has been developed.^[^ ^21^ ^]^ Compared with the direct encapsulation of drugs with nanoparticles (such as ZIF‐8), this self‐assembled nano‐prodrug exhibited higher encapsulation efficiency and drug‐loading stability. The experimental results showed that this nano‐prodrug exerted a synergistic effect of LND and NLG919 to simultaneously inhibit the accumulation of lactate and kynurenine in TME, thereby reinvigorating antitumor immunity. Of note, the effect of glycolytic inhibition on extracellular lactate regulation would be weakened by the adaptive evolution of tumor cells. To completely exhaust extracellular lactate, Tian et al. constructed a dual lactate flux‐interfered nanosystem (L@S/L) by incorporating glycolytic inhibitor LND and MCT4 inhibitor syrosingopine into liposomes to simultaneously inhibit lactate production and efflux.^[^ ^166^ ^]^ It has been proved that L@S/L promoted M1 phenotype polarization, increased NK cell recruitment, and decreased the frequency of immunosuppressive T_reg_ cells, hence driving the transition of “cold‐to‐hot” tumors. For extracellular lactate regulation, the targets of inhibition of lactate production and efflux are normally located in tumor cells or even subcellular organelles. By contrast, the strategy of promoting lactate degradation possesses easily accessible target sites that are distributed throughout the tumor tissues. Given this, some immunomodulatory nanosystems with LOX‐like activity have been developed for enhanced tumor immunotherapy.^[^ ^110^, ^113^, ^116^, ^117^ ^]^

It is worth noting that the acidic microenvironment caused by lactate accumulation should be partially responsible for tumor immune suppression.^[^ ^6^, ^10^ ^]^ Immune cells can recognize and respond to extracellular H^+^ through pH sensors on the cell surface.^[^ ^167^ ^]^ For example, the pH‐sensing G protein‐coupled receptors are ubiquitously expressed by immune cells and can be activated by the protonation of several histidine residues of these receptors;^[^ ^168^ ^]^ acid‐sensitive ion channels have been demonstrated to be expressed in monocytes, macrophages, and DCs and involved in acidosis‐mediated immune function impairment.^[^ ^169^ ^]^ Overall, antitumor effector cells (such as T cells and NK cells) tend to lose function and undergo an anergic state followed by apoptosis when exposed to a low pH microenvironment. Conversely, immunosuppressive cells, such as tumor‐associated macrophages, MDSCs, and T_reg_ cells, are induced by the acidic microenvironment to promote tumor growth while blocking antitumor immune responses. Therefore, neutralizing tumor acidity is beneficial for restoring antitumor immunity. Gillies et al. demonstrated that oral bicarbonate could boost the immunotherapeutic efficacy of anti‐PD1, anti‐CTLA‐4, or adoptive T‐cell transfer.^[^ ^170^ ^]^ To reduce the risk of alkalosis and improve the efficiency of pH regulation, some “alkaline” nanoparticles have been utilized for remodeling immunosuppressive TME to improve the response rate to immunotherapy.^[^ ^154^, ^171^ ^]^ For instance, Gu's group developed a CaCO_3_‐contained sprayed immunotherapeutic gel to inhibit postoperative tumor recurrence. Briefly, the anti‐CD47 antibody‐encapsulated CaCO_3_ nanoparticles (aCD47@CaCO_3_) incorporated fibrinogen solution and thrombin solution were sprayed and mixed at the surgical wound site to form an immunoregulatory gel in situ (Figure 14C).^[^ ^164^ ^]^ CaCO_3_ nanoparticles gradually dissolved in the TME to scavenge H^+^ and released anti‐CD47 antibodies, promoting M1‐type macrophage activation and antitumor T cell response, thus inhibiting tumor recurrence and metastasis (Figure 14D). Recently, a growing body of evidence suggests that using nanoparticles to alkalinize the TME can improve tumor immunotherapy.^[^ ^172^ ^]^

The combination of tumor lactate metabolic regulation with immunotherapy has attracted increasing attention as a candidate strategy for achieving effective antitumor therapy. However, most studies focus on temporary lactate regulation, with little attention paid to prolonging the lactate regulation time window, which may be more beneficial for the long‐term maintenance of the immune activity microenvironment.

### 3.3 Combining Lactate Metabolic Regulation with PTT

PTT, which utilizes photothermal transduction agents (PTAs) to induce localized high temperatures to ablate tumors, is a spatiotemporally controllable and noninvasive therapeutic approach.^[^ ^11^ ^]^ An ideal PTA should accumulate more in tumors than in surrounding normal tissues, reducing undesired adverse effects and improving PTT outcomes. Nano‐PTAs (e.g., noble metal materials, MOFs, carbon‐based nanomaterials) have piqued the interest of researchers due to their ability to accumulate in tumors via EPR effects and active targeting, and have promoted the development of PTT.^[^ ^1^ ^]^ However, thermotolerance ubiquitously exists in tumor hyperthermia because the expression of heat shock proteins (HSPs, such as HSP70, HSP90, and HSP110) elevates rapidly during thermal treatment.^[^ ^173^ ^]^ It is worth emphasizing that, as an indispensable energy currency for living organisms, ATP is required for HSP synthesis.^[^ ^45^ ^]^ Therefore, interference with lactate metabolism resulting in intracellular ATP depletion can block HSP synthesis and thus contribute to the enhancement of PTT efficacy.

**Figure 15:**
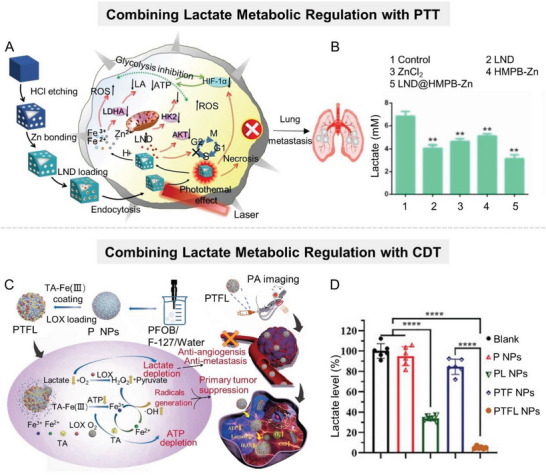
A) Scheme of the synthesis process of LND@HMPB‐Zn and its synergistic mechanism for glycolytic inhibition and PTT. B) Lactate concentration in the culture medium of B16 cells in different treatment groups. Reproduced with permission.^[^ ^30^ ^]^ Copyright 2022, Elsevier. C) Scheme of the synthesis of PTFL nanoparticles and their synergistic mechanisms for lactate metabolic regulation and CDT. D) Lactate level in the culture medium of 4T1 cells in different groups for 48 h. Reproduced with permission.^[^ ^178^ ^]^ Copyright 2021, Wiley.

Based on various developed nano‐PTAs with excellent properties, researchers have constructed a series of integrated nanosystems to combine lactate metabolic regulation with PTT.^[^ ^59^, ^72^, ^174^ ^]^ Simply put, lactate metabolism blockers are loaded into nano‐PTAs to impede ATP synthesis, thereby inhibiting HSP expression in tumor cells during PTT. For instance, Meng et al. utilized zinc‐enriched hollow mesoporous Prussian blue (HMPB‐Zn) nano‐PTAs loaded with the glycolysis inhibitor LND to construct a nanosystem (LND@HMPB‐Zn), which possessed both glycolysis inhibition and photothermal conversion properties (**Figure** **15**A).^[^ ^30^ ^]^ In addition to raising the local temperature of the tumor, LND@HMPB‐Zn‐mediated photothermal conversion also accelerated zinc ion dissociation and LND release, inhibiting LDHA and HK2 to downregulate glycolysis, respectively. The resulting reduction in lactate anabolism inhibited the synthesis of ATP and HSPs, resulting in enhanced PTT (Figure 15B). Additionally, Au‐based PTAs (e.g., GNRs and gold nanostars) have also been widely used in combination with lactate metabolism inhibitors to achieve efficient PTT due to the advances in synthesis, finely tuned absorption, easy surface modification, and high biosafety.^[^ ^11^, ^26^, ^59^ ^]^ On the other hand, targeted consumption of intratumoral lactate, an important fuel for tumor cell energy metabolism, may be a promising option to enhance PTT efficacy.

### 3.4 Combining of Lactate Metabolic Regulation with ROS‐Related Therapies

ROS play an important role in various biological processes of organisms, but an abnormal increase in intracellular ROS can cause cell death, mainly due to irreversible oxidative damage to key biomacromolecules such as lipids, proteins, and DNA.^[^ ^175^ ^]^ With the rapid progress in nanotechnology, a large number of nanomaterials capable of mediating ROS production have been developed to promote ROS‐induced toxic therapies, such as CDT, PDT, RT, sonodynamic therapy, etc. However, abundant endogenous antioxidants in tumor tissues can scavenge ROS, resulting in tumor resistance to ROS‐related therapies.^[^ ^176^ ^]^ Published studies have confirmed that lactate possesses antioxidant properties, and tumor resistance to ROS‐related therapies exhibits a positive correlation with intratumoral lactate levels.^[^ ^177^ ^]^ Therefore, decreasing intratumoral lactate levels is expected to overcome tumor resistance to ROS‐related therapies.^[^ ^97^, ^178^ ^]^

In addition, the deficiency of catalytic substrates (e.g., O_2_, H_2_O_2_) greatly limits the production of the therapeutic ROS, resulting in poor therapeutic outcomes.^[^ ^179^ ^]^ For example, CDT is a tumor treatment strategy that generates hydroxyl radicals (•OH) using H_2_O_2_ as a substrate, inducing tumor cell apoptosis. However, the tumor endogenous H_2_O_2_ level is too low (50 × 10^−6^–100 × 10^−6^ M) to provide an adequate substrate for the efficient Fenton reaction, thus limiting the yield of •OH and discounting the antitumor effect of CDT.^[^ ^49^, ^180^ ^]^ Considering that lactate can be oxidized to produce H_2_O_2_, promoting lactate catabolism can improve the therapeutic effect of H_2_O_2_‐dependent CDT.^[^ ^181^ ^]^ Therefore, some cascaded catalytic nanosystems have been developed for lactate metabolic regulation/CDT by loading LOX into nanoparticles with Fenton activity, such as those rich in iron, copper, or manganese.^[^ ^97^, ^178^, ^182^ ^]^ Typically, Tian et al. constructed a cascade catalytic nanosystem by coating a tannic acid (TA) ‐Fe (III) complex layer loaded with LOX on a perfluorooctyl bromide (PFOB) nanodroplet (Figure 15C).^[^ ^178^ ^]^ The O_2_‐sufficient PFOB nanodroplet promoted the LOX‐catalyzed oxidation of lactate to yield H_2_O_2_. Meanwhile, iron‐enriched TA‐Fe(III) complexes effectively induced the conversion of H_2_O_2_ to •OH. As a result, this cascade catalytic nanosystem could overcome insufficient endogenous substrates in the catalytic reaction, depleting lactate and ATP, and achieving efficient CDT (Figure 15D). In addition, a large number of similar studies have shown that the combination of lactate metabolic regulation and PDT can produce significant efficacy in tumor treatment.^[^ ^63^, ^77^, ^100^, ^131^, ^144^, ^183^ ^]^

### 3.5 Others

In addition to the common treatment modalities, lactate metabolic regulation is also beneficial in some novel therapies, including starvation therapy,^[^ ^70^, ^146^ ^]^ chlorine therapy,^[^ ^133a^ ^]^ and virotherapy.^[^ ^94^, ^184^ ^]^ For example, nanosystems targeting multiple tumor energy metabolism pathways have been developed to achieve efficient starvation therapy. Shi's group constructed a double‐gate controlled “energy interrupter” by loading DNAzymes into zinc‐enriched ZIF‐8 nanoparticles.^[^ ^185^ ^]^ This “energy interrupter” exhibited a dual inhibitory effect on metabolism, including 1) Zn^2+^‐overloading induced NAD^+^ decrement and GAPDH inactivation, inhibiting glycolysis, and 2) activated DNAzymes cleaved GLTU1 mRNA, cutting off glucose supply. In another example, a lactate‐regulating and ClO_2_‐loaded nanoplatform was constructed to regulate lactate signal channels for cascade amplification and enhancement of multimodal chlorine therapy.^[^ ^133a^ ^]^

In conclusion, the versatility of nanomaterials allows for the efficient integration of lactate metabolic regulation and other antitumor modalities into one nanoplatform and improved drug enrichment at the tumor site.^[^ ^186^ ^]^ These multifunctional nanoplatforms interfere with the energy metabolic process and break down tumor defense barriers by regulating lactate metabolism, thus overcoming tumors’ tolerance to treatments, such as immunotherapy, chemotherapy, PTT, and so on. Therefore, the multifunctional nanoplatforms targeting lactate metabolism are conducive to the exploration of more effective antitumor therapy strategies.

## 4 Challenges in Clinical Transformation

Although extensive studies have shown that nanomaterials hold great potential in tumor therapy, they are still confronted with significant challenges in clinical transformation, which significantly impede the clinical application of nanomedicine. These challenges fundamentally arise from biosafety, delivery efficiency, reproducible synthesis, scalable manufacturing, storage, and transportation,^[^ ^187^ ^]^ which should be considered when designing rational antitumor nanomedicines, including those that interfere with lactate metabolism.

Biosafety is the first challenge for clinical transformation of nanomedicine. The biosafety of nanoparticles in vivo is largely influenced by their physicochemical properties, including particle shape, size, surface modification, surface charge, chemical properties, components, stability, and so on.^[^ ^188^ ^]^ These factors will affect cellular uptake, interaction with biological macromolecules, biodistribution, biodegradation, and metabolic clearance, resulting in specific biological effects. After injection into the body, the stimulation caused by nanoparticles or their degradation products may induce a certain degree of inflammatory response, resulting in cellular and tissue damage. Even if they do not cause obvious acute toxicity, their long‐term retention in the organism is a serious concern, as it can lead to oxidative damage, inflammation, and fibrosis.^[^ ^189^ ^]^ Although several reported antitumor nanomedicines, including lactate‐regulating nanomedicines, have demonstrated satisfactory biosafety, their long‐term toxicity remains to be evaluated. Moreover, most current research on nanoparticle biosafety is still superficial, with published data remaining in cell culture or animal experiments stages.^[^ ^190^ ^]^ Currently, issues, such as side effects and toxicity of nanoparticles, remain controversial, and these risks must be thoroughly considered prior to application to human system.

Systemically administered nanoparticles would experience multiple biological barriers before reaching the target sites, resulting in reduced delivery efficiency, which is another challenge for clinical transformation of nanomedicines.^[^ ^191^ ^]^ Although nanotechnology holds great promise for targeted drug delivery, enrichment of nanoparticles within target tumor cells remains severely insufficient. After entering the blood circulation, the majority of nanoparticles would be identified, captured, and cleared by mononuclear phagocyte system. Various strategies have been developed to improve the delivery efficiency of nanoparticles, including surface modification with a protective layer (e.g., PEG, HA, etc.), shape and size optimization, introduction of targeting ligands, etc.^[^ ^1^ ^]^ It should not be ignored that surface modification may also reduce the interactions between nanoparticles and tumor cells. Furthermore, the transport of therapeutic agents to solid tumors is not the end point of drug delivery. Nanoparticles still need to penetrate the extracellular matrix (ECM) of tumors and enter the targeted tumor cells to exert antitumor effects. Due to aberrant vascular networks, elevated interstitial fluid pressure, and dense ECM, nanoparticles must overcome considerable transport barriers to achieve deep tumor penetration.^[^ ^192^ ^]^ It is well known that diffusion rate is inversely proportional to particle size, so nanoparticle size is crucial for tumor penetration. However, the pharmacokinetics and tumor enrichment of ultrasmall nanoparticles are usually not ideal. Therefore, to successfully deliver sufficient quantities of nanoparticles into solid tumors for efficient lactate metabolic regulation or other therapeutic effects, multiple biological barriers need to be considered simultaneously when it comes to nanomedicine design.

Reproducible synthesis and scalable manufacturing are essential for the transition of nanomedicine from laboratory to clinical development and subsequent commercialization.^[^ ^193^ ^]^ Usually, preclinical and early‐stage clinical trials are conducted with small amounts of nanomaterials. In large‐scale production, variations in the physical and chemical properties of nanoparticles from batch to batch may occur due to the polydispersity and synthesis complexity of nanoparticles.^[^ ^187^ ^]^ Therefore, the industrial manufacturing of antitumor nanomedicines requires strict control of their physicochemical properties, which makes the chemistry, manufacturing and control process and good manufacturing practice requirements more demanding. Furthermore, reproducible synthesis and scalable manufacturing will be extremely difficult when nanoparticle formulations involve complex techniques or multiple steps. Therefore, the complexity of synthesis must be considered in early nanomedicine design.

In addition, nanoparticle bioapplications typically employ liquid suspensions, which makes their storage and transportation challenging. The instability of nanoparticle suspensions stems from aggregation or fusion, hydrolysis, premature drug leakage, and so on. Therefore, maintaining the initial physicochemical state of antitumor nanomedicines properly until their final clinical application is of great importance for commercialization.

In conclusion, a successful clinical transformation of nanoparticles requires the development of a safe, simple, and cost‐effective synthesis mode, as well as a better understanding of nanoparticle biodistribution, pharmacokinetics, and safety mechanisms.

## 5 Conclusions and Outlook

Lactate accumulation is a representative feature of TME. Numerous studies have demonstrated that tumor‐derived lactate shapes a protumor microenvironment, which promotes tumor proliferation and invasion, angiogenesis, immune escape, and resistance to therapies (including chemotherapy, immunotherapy, PTT, ROS‐related therapies, etc.),^[^ ^6^, ^149^, ^163^, ^177^, ^199^ ^]^ leading to poor therapeutic outcomes and prognosis. Therefore, interfering with tumor lactate metabolism is of great significance for exploring new antitumor strategies and improving the effectiveness of the existing antitumor therapies. With the advancement of modern molecular biology, structural biology, combinatorial chemistry, and genetic engineering, small‐molecular and biomacromolecule antitumor drugs have entered a stage of rapid development. At present, various small‐molecule inhibitors and gene agents targeting tumor lactate metabolism have emerged with considerable advantages in tumor therapy.^[^ ^200^, ^202^, ^203^, ^204^, ^205^, ^206^, ^207^ ^]^ However, these agents share common shortcomings, including poor circulatory stability, low bioavailability, poor accessibility to tumors, and undesirable off‐target effects, which greatly hinder their clinical translation. Recent years have witnessed explosive development and innovation in nanoscience and nanotechnology, which is revolutionizing antitumor drug delivery. It has been intensively proven that nanoformulations are capable of optimizing the pharmacokinetics of lactate metabolism‐targeting agents to significantly improve bioavailability and reduce toxic side effects, and realize the integration of tumor diagnosis and treatment.^[^ ^194^ ^]^ More importantly, nanomaterials have exhibited several additional advantages that facilitate antitumor therapy based on lactate metabolic regulation, including the following.

1. Most nanomaterials can readily codeliver two or more drugs. Due to the heterogeneity and plasticity of tumor cells, the therapeutic effect of lactate metabolic regulation via a single target is very limited. Multidrug combination therapy has been shown to intensively improve antitumor activity and reduce drug resistance.^[^ ^198^ ^]^ Notably, the success of combination therapies is built on the fact that all drugs function simultaneously in a fairly narrow space (e.g., individual cells) at an appropriate molar ratio. Nanotechnology can integrate multiple therapeutic agents with different water solubility and pharmacokinetic characteristics into one platform, ensuring that agents with diverse therapeutic functions share the same pharmacokinetic fate during in vivo circulation.^[^ ^29^ ^]^ Therefore, nanomaterials can maximize the efficacy of lactate metabolic regulation‐based multidrug combination therapies.
2. Nanomaterials can directly or indirectly interfere with lactate metabolism due to their inherent biochemical activities. For example, some nanoparticles (e.g., SeNPs) can directly block lactate production in tumor cells by inhibiting the activity of glycolytic‐related enzymes.^[^ ^88^ ^]^ Some nanoparticles (e.g., metal sulfides, oxygen‐producing nanoparticles) can indirectly interfere with lactate metabolism by triggering specific chemical reactions in tumors to produce regulatory molecules (e.g., H_2_S, O_2_).^[^ ^37^, ^139^ ^]^ Some nanoparticles (e.g., SnSe nanosheets, alkaline nanoparticles) can directly scavenge lactate or H^+^ in TME to reverse the tumor‐promoting microenvironment.^[^ ^110^, ^154^ ^]^
3. In addition to serving as carriers, many nanoparticles exhibit efficient tumor‐killing effects, such as Au nanoparticles, Prussian blue, Porphyrin MOF, Fe‐based nanoparticles, and so on. The intrinsic therapeutic functions of nanoparticles are advantageous in reducing the complexity of the integrated nanoplatforms, which promote the organic combination of lactate metabolic regulation and other therapeutic modes.^[^ ^26^, ^30^ ^]^ Due to the complexity and plasticity of tumor metabolism, single therapeutic strategies based on lactate modulation usually rarely result in efficient tumor‐killing effects. When one metabolic pathway is inhibited, tumor cells can develop other compensatory metabolic pathways via metabolic reprogramming to maintain metabolic homeostasis. For example, inhibiting MCT1 in MCT1‐overexpressed tumor cells with siRNAs or small‐molecule inhibitors may promote the transformation of oxidative tumor cells into glycolytic types.^[^ ^195^ ^]^ In addition, as previously stated, regulating lactate metabolism can place tumor cells in a metastable condition, increasing the sensitivity of targeted tumor cells to various therapeutic modalities. Therefore, regulating lactate metabolism while combining the therapeutic functions of nanoparticles in situ is of great importance for achieving efficient antitumor effects.

This review discusses several major strategies of nanomaterial‐mediated lactate metabolism modulation for oncotherapy in recent years, including inhibiting lactate anabolism, promoting lactate catabolism, and disrupting the “lactate shuttle”. To help appreciate these strategies, we cite some typical examples and discuss their benefits and drawbacks in depth. Despite the obvious benefits of lactate metabolic regulation in tumor suppression, targeting lactate metabolism alone exhibits limited antitumor effect, owing to its inability to directly kill tumor cells. To achieve better antitumor effects, researchers are vigorously exploring effective combined antitumor strategies that incorporate lactate interference with other therapeutic modalities, including chemotherapy, immunotherapy, PTT, and ROS‐related therapies.^[^ ^30^, ^155^, ^165^, ^178^, ^208^, ^209^, ^210^ ^]^ To accomplish this, a series of versatile nanoplatforms have been developed. These versatile nanoplatforms can realize the integration of multiple therapeutic components and promote the complementarity among different therapies to generate a superadditive (i.e., 1 + 1 > 2) therapeutic effect.^[^ ^196^, ^201^ ^]^ Although versatile nanoplatforms have demonstrated success in reversing protumor environments, overcoming therapeutic resistance, and improving tumor‐killing effects, the following issues need to be addressed to facilitate clinical translation.

1. Although integrated nanosystems can facilitate the complementarity of multiple therapies to overcome monotherapy flaws, it comes at the expense of increased nanosystem complexity. Furthermore, differences between therapeutic components within one nanosystem, including the location of targets and optimal time of action, may offset some of the optimized efficacy. Therefore, more research is needed to elucidate the transport and metabolic processes of nanosystems in vivo, guiding the reasonable nanosystem design to simplify nanosystem structure and maximize the therapeutic potential of each component.^[^ ^6^ ^]^
2. The lactate metabolism varies among tumors with different types, sizes, locations, and stages.^[^ ^6^, ^92^, ^142^ ^]^ Therefore, it is necessary to screen appropriate interfering agents and formulate more targeted therapeutic strategies according to specific situations, so as to achieve more precision therapy.
3. The risks that nanocarriers themselves pose to patients should not be overlooked. For instance, some non‐biodegradable nanoparticles may accumulate continuously in the body, eventually leading to chronic inflammation.^[^ ^197^ ^]^ In addition, the degradation, metabolism, and biochemical conversion behaviors of versatile nanosystems with multicomponent are extremely complex in vivo, which may cause unpredictable toxic side effects. Therefore, nanosystems need to undergo extremely rigorous safety evaluation before clinical translation.

Overall, despite the challenges in this field of lactate metabolic regulation for antitumor treatment, various nanosystems have emerged as effective and biocompatible candidates for improving bioavailability and clinical applicability. Through a comprehensive review and in‐depth analysis of numerous recent advances in this field, we hope to provide useful guidance for the future construction of high‐performance nanomaterials interfering with lactate metabolism and the development of fine multimodal nanoplatforms for intensive tumor therapy.

## Conflict of Interest

The authors declare no conflict of interest.
